# Comparative study on government subsidy models for competitive drug supply chains under centralized procurement policy

**DOI:** 10.3389/fpubh.2025.1542858

**Published:** 2025-03-14

**Authors:** Yan Wen, Yan Wei, Lu Liu

**Affiliations:** School of Business, Qingdao University, Qingdao, China

**Keywords:** centralized procurement, government subsidies, differential game, drug supply chain, innovative drug

## Abstract

As the generic drug market tends to be saturated, the structural transformation of generic drug companies is imminent, while the high investment and high-risk attributes of innovative drug research and development aggravate the transformation difficulties. Against the backdrop of drug centralized procurement policy, considering the effect of health insurance reimbursement and market competition ferocity, this study constructs a differential game model of a secondary drug supply chain comprising two competing drug companies and a single healthcare institution. In addition, this study comparatively analyzes the optimal equilibrium strategies and supply chain profit levels of drug research and development investment and healthcare service efforts under four government subsidy modes, further discussing them along with arithmetic examples. It is found that the government's subsidy behavior markedly influenced drug companies' investment in drug research and development and healthcare institution' service cost investment. Besides, different incentives for supply chain members' decision-making and profits were noted in different markets with different competition intensities. In the low-intensity competition market, the government's subsidies to innovative drug companies generate much higher social welfare than other modes. In the high-intensity competition market, the government subsidized healthcare institution can minimize the mutually exclusive effects of subsidies on the development of innovative and generic drug companies, and eventually drive the reform and development of the entire drug industry.

## 1 Introduction

To decrease the price of drugs and lessen the burden of medical care for the public, the National Health Insurance Bureau made centralized purchasing the dominant mode of drug procurement for public healthcare institutions, and endorsed the standardization and institutionalization of centralized purchasing (1). This has led consumers to generally choose drugs that are covered by medical insurance when making their purchasing decisions. In this context, if generic drugs fail to be included in the centralized procurement list, they will not only lose a significant market share in public hospitals and pharmacies, but also face severe market competition, which will greatly compress the profit margins of enterprises (2). Furthermore, the “China Generic Drug Development Report (2023)” showed a continuous decline in the market share of Chinese chemical generics, from 60% in 2018 to 52% in 2023 in the overall drug market, and from 79% to 74% in the chemical drug market. This indicates that although generics still account for a significant proportion, their market share is on the decline. In contrast, the market share of innovative drugs continues to increase, and the development of innovative drugs is the future trend for Chinese drug companies (3). Therefore, generic drug companies should undergo industrial transformation, enhance their independent innovation capabilities, and focus on the development of innovative drugs to drive the growth of their business performance (4). Innovative drug R&D is distinguished by high profits and high risks, rendering such projects highly challenging entailing massive investment and considerable return duration (5). From the project to the market, the process typically takes 5–10 years, with a success rate of < 10 % (6). The price premium is barely sufficient to cover the increase in R&D costs, thereby markedly decreasing the inclination of the drug companies to turn to innovative drugs, accounting for the absence of a large number of local brands of innovative drugs (7).

To kindle the innovation power of drug companies and safeguard the popularity of social medical benefits, the state has introduced several preferential policies, including the priority approval of innovative drugs, the priority declaration of generic drugs that have passed the consistency assessment to the National Health Insurance catalog, the protection of intellectual property rights of novel drugs, and the dynamic modification of the health insurance catalog, providing a favorable policy setting for the development of domestically produced innovative drugs and generic drug brands (8). Meanwhile, some local governments, such as Hubei province, have announced incentive policies to gain the national class I new drug production approval of companies, each product to receive a 30-million-yuan incentive, and has award 20% of the actual research and development costs of the first generic drug of its kind in the country that passes the consistency evaluation; likewise, Shandong Province has given 20-million-yuan of comprehensive post-subsidy funding support for class I new drugs with independent intellectual property rights that have been industrialized in the province. It is evident that government subsidies play a crucial role in promoting the development of domestic innovative and generic drug brands (9). Through policy interventions, the government can alleviate the financial pressures on enterprises during their transformation, incentivize them to enhance their innovation capabilities, thereby improving the quality of drugs and optimizing the market competition landscape (10).

In addition, healthcare institutions play an important role in the implementation of the centralized procurement policy, both in terms of the need to prioritize the use of winning drugs in accordance with the policy requirements and in terms of meeting procurement volume targets. At the same time, the government incorporates the procurement and usage performance into their evaluation criteria. This may limit the flexibility of healthcare institutions in choosing drugs based on clinical needs, thus reducing their enthusiasm for participation (11). Therefore, to incentivize the participation of healthcare institutions in the centralized purchasing policy, the government provides balance retention incentives for healthcare institutions that meet the usage standards through savings in health insurance funds (12). For example, in the 2022 Zibo City initiative for centralized procurement of drugs to rural health stations, the government provided surplus retention rewards to eligible health stations, totaling 863,000 yuan, with an average reward of 693 yuan per station. This measure not only promotes the rationalization of drug procurement but also facilitates the use of domestic generic drugs, thereby reducing overall social healthcare costs.

In summary, under the centralized procurement policy, government subsidies have extensively covered various segments of the drug supply chain. Through different subsidy models, the government has played a significant role not only in controlling drug prices and ensuring the supply of essential drugs, but also in achieving remarkable success in stimulating drug companies' research and development innovation. However, existing research primarily focuses on the exploration of single subsidy strategies, such as research funding, tax incentives, and price controls, with insufficient comparison of the effects of specific subsidy models in competitive drug supply chains. Therefore, researching which government subsidy strategies can more effectively promote the healthy development of the biomedical industry while driving reforms in public healthcare institutions is a question of significant theoretical and practical importance.

Hence, this study aims to investigate the following three questions:

(1) How do drug companies' innovation investment strategies, drug quality, and supply chain performance alter under different government subsidy policies? Do drug companies prefer high-margin innovative drug development or low-investment generic drug development?
(2) How do changes in Medicare reimbursement rates and competition intensity among drug companies influence the decisions of drug supply chain members and supply chain performance?
(3) In an increasingly competitive market setting, how can the government articulate subsidy policies that can better promote the healthy development of domestic innovative and generic drug brands, as well as promote the enhancement of the social welfare of the entire healthcare industry?

To address the three problems mentioned above, this study contemplates the impact of health insurance reimbursement and centralized drug procurement policy and constructs a differential game model of the secondary drug supply chain comprising competing drug companies and healthcare institution, considering four different government subsidy models: no government subsidy (NS); subsidy for innovative drugs (IS); subsidy for generic drugs (GS); subsidy for healthcare institution (HS). In addition, this study analyzes the effects of different government subsidy models and market competition intensity on drug quality, R&D strategies, and profit levels of drug supply chain members, and further discusses them with arithmetic examples.

This study offers certain theoretical research value and practical value. Regarding theoretical research, it enriches the research on government-guided product transformation of drug companies, as well as expands the research field related to government subsidies in the drug supply chain. Its practical significance is that when the government subsidizes R&D in the drug supply chain, both drug companies and healthcare institutions can profit from it, thereby improving the socioeconomic benefits. Furthermore, this study provides certain references for drug supply chain members on how to amplify their economic benefits and fulfill their social responsibilities with the assistance of government subsidies.

The rest of the paper is organized as follows: in Section 2, we summarize the relevant literature; Sections 3 and 4 present the relevant models and analyze the optimal equilibrium strategies under each subsidy model; Section 5 presents the analysis of the model results; Section 6 provides numerical analyses of the key parameters in order to draw further conclusions; and in Section 7, the conclusions drawn in this paper are summarized and important implications are discussed.

## 2 Literature review

In this section, we review relevant literature from the following three streams: government subsidies in the drug supply chain, drug innovation, and competitive supply chains.

### 2.1 Government subsidies in the drug supply chain

In the field of research on government subsidies in the drug supply chain, the existing literature predominantly focuses on subsidies for drug companies and subsidies for healthcare institutions.

In terms of subsidies for drug companies, some scholars argued that government subsidies have a positive impact on companies' innovations, and that market participants who receive government subsidies tend to generate more profits and greater social welfare (13, 14) while the game between the government, companies, and their competitors determines how subsidy policies impact companies' innovation (15, 16). Yang and Xu (17) found that government R&D subsidies have a significant positive effect on the innovation performance of drug companies, whereas tax incentives were found to have no significant positive effect on innovation performance. Furthermore, Lanahan et al. (18) and Kleine et al. (19) observed that, in addition to government subsidies, firm heterogeneity can also enable companies with varying production capacities to pursue various innovation strategies. Meanwhile, government subsidy programs often have a significant impact on technologically innovative companies (20). Chen et al. (21) constructed a two-stage dynamic game model and found that under different subsidy strategies, drug revenues were positively correlated with the amounts of R&D subsidies per unit of product. Xue et al. (22) compared the impact of government subsidies in the drug companies' R&D decision-making and profits of drug companies under the horizontal and vertical spillovers effects.

In terms of subsidies for healthcare institutions, Zhao and Zhang (23) suggested that government revenue subsidies play a crucial role in alleviating the reform dilemma of public healthcare institutions. Chen et al. (24) found that government subsidies reduce the burden of healthcare costs on patients and enhance the cost efficiency of healthcare institutions. Hua et al. (25) examined how government subsidies coordinate the healthcare system to maximize social welfare under competition between private and public healthcare institutions.

Influenced by the centralized drug procurement policy and the zero-differential rate policy, the drug supply chain distribution model differs from other supply chains, while all of the above studies have ignored the impact of the policies in practice on the structure of supply chain drug sales. For this reason, this study constructs a government subsidy model based on the actual structure of the drug supply chain. It aims to reflect the decision-making and profit changes of the supply chain members under the real subsidy environment, in order to provide more practical decision-making insights for the transformation of product R&D in drug companies and the reform of the revenue structure in healthcare institutions.

### 2.2 Drug innovation

Making substantial investments in research and development is an integral part of the drug innovation process, which also affects the profitability of drug companies. Although Curtis et al. (26) argued that R&D investment gradually reduced the level of profit volatility of drug companies, Parra (27) showed that drug companies can still increase their profit level by increasing their R&D investment in drugs during the period of drug patent protection. The existing literature examines the factors influencing drug companies' R&D and innovation, including health insurance reimbursement (28), market size (29), centralized purchasing (10, 30), and drug prices (31, 32). Among these studies, Zhang and Nie (28) conducted a natural experiment based on China's New Cooperative Medical Scheme (NCMS) to study the impact of drug companies' innovation for diseases covered by the NCMS, with the results demonstrated that the government's implementation of the health insurance policy effectively incentivized drug companies to develop new technologies. Pierre et al. (29) quantitatively analyzed the relationship between drug market size and enterprise R&D, and found a mutually reinforcing effect between the two. Chen et al. (10) used data from China's A-share listed drug companies in Shanghai and Shenzhen from 2016 to 2022, and applied a double-difference model to empirically examine the impact of the centralized banded purchasing policy on drug companies innovation, and further analyzed its mechanism of action. Meanwhile, Ke et al. (30) examined in depth the impact of the centralized drug procurement policy on the business performance of winning companies and how these companies can adjust their business decisions from both theoretical and empirical perspectives. Furthermore, Hammoudeh and Nain (31) demonstrated that there is a significant difference in the pricing strategies of highly innovative drug companies compared to others. While the former increased drug prices, there is also evidence that the drug prices of less innovative companies generally decreased after mergers.

Most existing studies explore the impact of drug R&D innovation on profits through qualitative analysis, and only a few studies have employed static quantitative methods, which overlook the effect of drug companies' investment in R&D innovation on drug quality and long-term profits. Furthermore, most research focuses on innovative drug companies, while research on drug innovation in the large number of generic drug companies in China remains limited. Thus, with the full implementation of the centralized drug procurement policy, how to innovate products, maintain the competitiveness of the drug market, and enter the national drug centralized procurement catalogs has become a critical issue that many generic drug companies need to urgently solve. This study employs differential game theory to combines government subsidies, medical insurance reimbursement and other drug policies, investigating the drug R&D and innovation strategies of both innovative and generic companies under the centralized procurement policy from a dynamic perspective, which better reflects the real needs of companies and is more representative of the industry.

### 2.3 Competitive supply chain

In the field of competitive supply chain research, the existing literature primarily focuses on competition among manufacturers, retailers, and in dual-chain supply chains. Li and Li (33) examined the effects of decision order, price and quality competition on the supply chain game equilibrium and members' profits. Zhao et al. (34) found that the manufacturer's competitive environment generates consistently higher profits for product pricing choices created through synergistic cooperation between the two parties, and Chen et al. (35) confirmed similar findings regarding channel choices for manufacturers producing competing products. As the intensity of competition increases, Liu and Huang (36) found that manufacturers' R&D investment decreases with the increase of R&D spillovers. Consequently, Johari and Hosseini-Motlagh (37) established a corporate social responsibility (CSR) cost-sharing contract and cost burden reduction for drug companies in terms of drug pricing and CSR. Deng et al. (38) explored the impact of supply chain competition on product sustainability and profitability, and demonstrated that competition among sustainable products plays an important role in determining firm decisions. Wu et al. (39) investigated the effects of spillover rate, R&D efficiency and level of competition on the equilibrium scenario of R&D competition and cooperation under manufacturer competition. Wang et al. (40) found that consumers' green preferences and competition among manufacturers contribute to optimal pricing and retailer profits, but under certain conditions, it can harm manufacturers' utility and supply chain profits. In contrast, Zhou et al. (41) compared the effects of price competition and simultaneous price and service competition on members' profits by constructing a network competition model of a product-service supply chain containing multiple competing manufacturers and retailers. Zheng et al. (42) examined the effects of competitive intensity and product recall on optimal price, profit, and decision choices in a supply chain consisting of two manufacturers and two retailers. Finally, Feng and Liu (43) investigated the selection strategies of green product R&D in two green supply chain competitions, as well as the effects of price competition and green R&D costs on sustainable competitive supply chain prices, green levels and corporate profits in a sustainable competitive supply chain.

To summarize, based on the drug supply chain comprising two competing drug companies and a healthcare institution, this paper combines the actual policies of centralized drug procurement and medical insurance reimbursement to study the R&D and innovation strategies of drug companies and the service input strategies of healthcare institutions under the different governmental subsidy modes. At the same time, the impact of real factors such as subsidy level, competition intensity, and health insurance reimbursement policy are fully taken into account, in order to explore the optimal decision-making and profit maximization of drug companies and healthcare institutions, and to more comprehensively reflect practical realities and provide guidance for practice.

## 3 Problem description and assumptions

Given the presence of multiple competing drug companies in reality, such as patients with influenza-related drug needs, who may choose between the original oseltamivir produced by Roche and the generic version manufactured by Sunshine Lake Pharma, which has passed consistency evaluation, a competitive drug supply chain model consisting of two competing drug companies and a single healthcare institution is constructed. This study analyzes and compares the optimal feedback strategies of drug companies' R&D investment and healthcare institution' service efforts under four different scenarios, along with the impacts of different subsidy models and competitive intensities on drug quality, supply chain performance, and overall social welfare.

### 3.1 Model description

**Figure 1:**
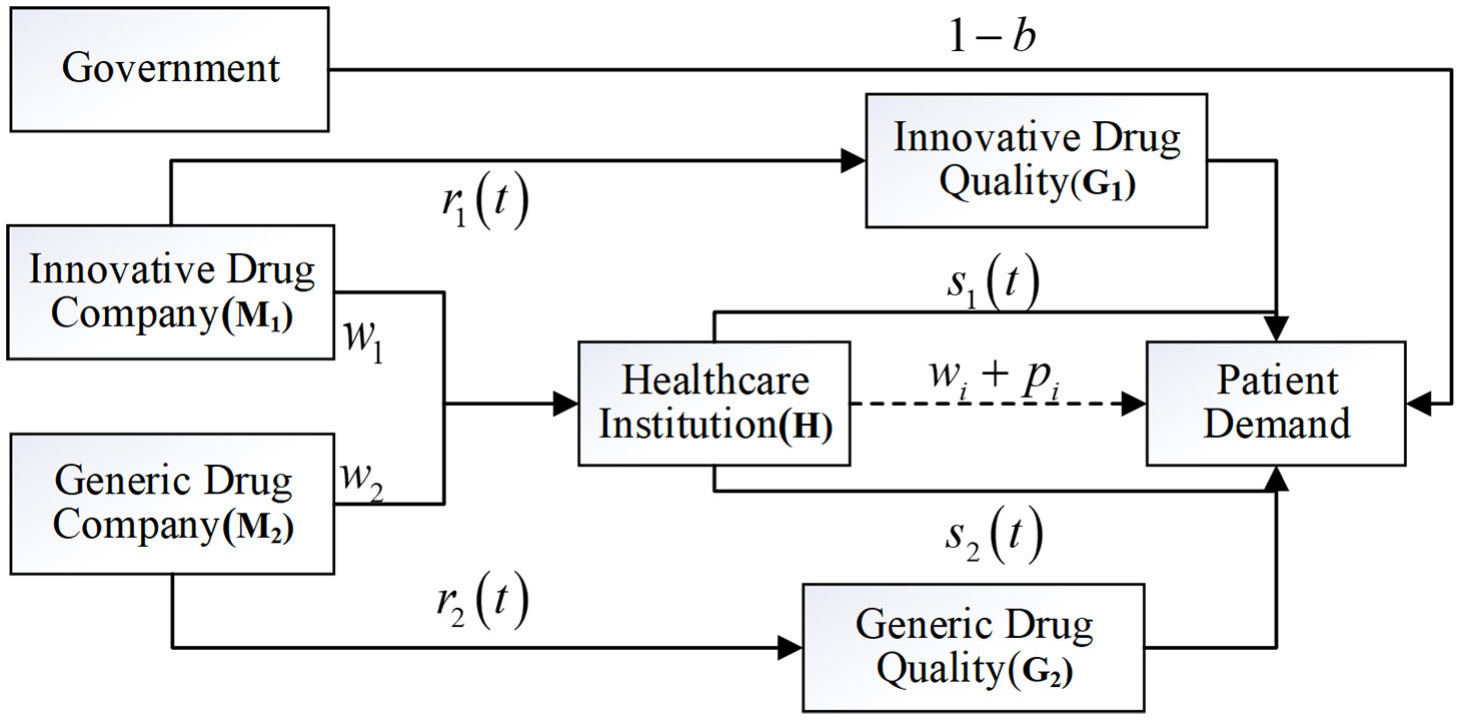
Model of government subsidies in competitive drug supply chains.

Competitive drug supply chain consisting of two drug companies M_1_, M_2_ and healthcare institution H (Figure 1), drug company M_1_ develops patented innovative drugs, while drug company M_2_ produces generic drugs with the same therapeutic effect. Both drugs have passed the consistency evaluation and are included in the national health insurance catalog, and are sold to the patients through the same healthcare institution H, allowing patients to enjoy the same health insurance reimbursement benefits when purchasing these drugs. In this system model, innovative drug companies consider the impact of decision-making on long-term interests, i.e., the dynamic changes in drug quality, while generic drug companies only consider the impact of decision-making on current interests and do not account for these dynamic changes in drug quality. Furthermore, to incentivize technological development of innovative drug companies and the industrial transformation in generic drug companies, the government proposes financial subsidies to supply chain members to promote the rapid development of domestic innovative and generic drug brands. In response to the government's different subsidy options, four subsidy models are composed as follows:

(1) NS subsidy model. As the baseline model, neither drug companies nor healthcare institution receive subsidies from the government;
(2) IS subsidy model. Under this model, the government aims to stimulate drug innovation and promote domestically produced innovative drug brands by providing research and development incentive funds to entities producing innovative drugs that have passed Phase I-III clinical trials;
(3) GS subsidy model. Under this model, the government encourages generic drug R&D and reduces medical costs for patients by granting incentive funds to entities producing generic drugs that have passed the consistency evaluation for quality and efficacy;
(4) HS subsidy model. This model supports the scaling up of domestically produced generic drugs and reduces overall medical costs by encouraging healthcare institutions at all levels to prioritize the use of generic drugs that have passed the consistency evaluation, providing assessment incentives to those institutions meeting the required standards.

The symbols and meanings of key parameters and decision variables are presented in Table 1.

**Table 1:** Parameters and meanings.

**Parameters (*i* = 1, 2)**	**Meanings**
*w*_*i*_	Drug margins for drug companies
*p*	Marginal profit on services of healthcare institution
μ	Influence factors of R&D innovation investment on drug quality
*k*	Natural decay rate of drug quality
*a*	Initial size of the drug market
ε	Willingness of healthcare institution to recommend drugs
α, β, λ	The influence factors of drug research and development investment, service effort of healthcare institution and drug quality on demand, respectively
*d*	Intensity of competition in the market
*b*	Percentage of patients' out-of-pocket prices for drugs
ρ	Discount rate
θ	Proportion of R&D input cost subsidy for innovative drug enterprises
δ	Proportion of R&D input cost subsidy for generic drug enterprises
η	Unit centralized price subsidy ratio for generic drugs in healthcare institution
**Decision variables**
*r*_*i*_(*t*)	Level of drug companies' investment in drug R&D
*s*_*i*_(*t*)	Level of service inputs in healthcare institution
**State variable**
	Goodwill of innovative and generic drug brands

### 3.2 Model assumptions

**Assumption 1:** In the process of centralized drug procurement, drug company M_1_ determines the marginal profit *w*_1_ of innovative drugs, and drug company M_2_ determines the marginal profit of generic drugs. Considering that innovative drugs require significant time and financial investment during the development process, while offering superior efficacy and higher safety, and that innovative drugs enjoy market exclusivity during their patent period, whereas, generic drugs face intense competition upon entering the market, leading to a significant reduction in their prices (44), we assume that *w*_1_>*w*_2_. Healthcare institution purchase drugs from drug companies and sell them to patients in accordance with the “zero-differential-rate” policy, and are permitted to charge only for medical services such as registration fees (45). The price of medical services of healthcare institution does not change according to the difference of drugs, which determines the marginal profit of the services of healthcare institution *p*. Drug companies and healthcare institution have the same time discount rate ρ, and both of them aim at maximizing their own interests.

**Assumption 2:** The R&D inputs of drug companies M_1_ and M_2_ are denoted as *r*_1_(*t*) and *r*_2_(*t*), respectively, and the service inputs of healthcare institution H for the two drugs are denoted as *s*_1_(*t*) and *s*_2_(*t*). Based on the assumptions of Li and Chen (46) and Zhang and Hezarkhani (47), the R&D input cost of drug companies and the service input cost *C*_H_(*t*) of healthcare institution are


(1) $$\{\begin{array}{l} C_{\text{Mi}}(r_{i}(t))=\frac{1}{2}\kappa_{\text{Mi}}r_{i}^{2}(t),\;i=1,2\\ C_{\text{H}}(s_{i}(t))=\frac{1}{2}\kappa_{\text{H}}s_{i}^{2}(t),\;i=1,2 \end{array}$$

where κ_Mi_, κ_Hi_>0, *i* = 1, 2 denotes the R&D input cost coefficients and service cost coefficients, respectively, and for convenience of computation in this paper the which is normalized to 1.

**Assumption 3:** Drug quality is determined by the drug company's R&D investment and will decay over time, the Nerlove-Arrow (48) model is used to describe the process of change in drug quality, so the equation of state of drug quality is assumed to be:


(2) $$\{\begin{array}{l} {\dot{G}}_{i}(t)=\mu r_{i}(t)-kG_{i}(t)\\ {\dot{G}}_{i}(0)=G_{0},\;i=1,2 \end{array}$$

μ>0 denotes the coefficient of influence of R&D inputs on the quality of drugs, *k*>0 denotes the natural attenuation factor,*G*_0_ denotes initial goodwill.

**Assumption 4:** Patients' choice of drugs is often influenced by the medical staff's recommendations, and there are differences in their willingness to use different drugs (49), which can affect the market demand for drugs to a certain extent. Additionally, when patients choose drugs, they can also consider the drug price, health insurance reimbursement, the scientific and technological strength of drug companies and the quality of drugs and other factors. Referring to the assumptions of Ma et al. (50), the demand function of drugs is as follows:


(3) $$\{\begin{array}{ll} Q_{1}(t) & =\varepsilon a-bw_{1}+\alpha r_{1}\left(t\right)+\beta s_{1}\left(t\right)+\lambda G_{1}\left(t\right)\\ \; & +d\left(r_{1}\left(t\right)-r_{2}\left(t\right)\right)\\ Q_{2}(t) & =\left(1-\varepsilon \right)a-bw_{2}+\alpha r_{2}\left(t\right)+\beta s_{2}\left(t\right)+\lambda G_{2}\left(t\right)\\ \; & +d\left(r_{2}\left(t\right)-r_{1}\left(t\right)\right) \end{array}$$

where *a*>0 is the market size, ε>0 represents the proportion of healthcare institution recommending patients to use innovative drugs, *b*>0represents the proportion of patients' out-of-pocket prices for purchasing drugs, α>0, β>0, and λ>0 reflect the coefficients of the impact of R&D and innovation inputs, medical services efforts and drug quality on demand, and *d*∈(0, 1) represents the coefficient of the impact of the difference in R&D and innovation inputs of the two drugs on the demand, i.e., the intensity of competition in the market.

**Assumption 5:** To incentivize the technological development of innovative drug companies, the government provides subsidies to innovative drug R&D entities covering a proportion of their R&D costs, denoted as θ>0. Simultaneously, to ensure the successful transformation of products by generic drug companies, the government provides subsidies for the proportion of input costs δ>0 for the innovative development of generic drug products. In addition, in order to encourage the brand development of domestically produced generic drugs, the government subsidizes healthcare institution using domestically produced generic drugs with unit drug price η*w*_2_, where η>0 is the proportion of subsidies and *w*_2_ is the centralized procurement price of generic drugs. The government bases its subsidy decision mainly on maximizing social welfare, which includes manufacturer's profit π_M1_, healthcare institution's profit π_M2_, and consumer's surplus *CS*. The social welfare function is expressed as follows:


(4) $$SW=\pi_{\text{M1}}+\pi_{\text{M2}}+\pi_{\text{H}}+CS$$

Refer to Panda et al. (51) for the calculation of consumer surplus.


(5) $$CS=\int_{P_{\min}}^{P_{\max}}D\left(p\right)\text{ d}p=\int_{\left(a-D\right)/\tau }^{a/\tau }\left(a-\tau p\right)\text{d}p=\frac{D^{2}}{2\tau }$$

D denotes the demand function of the market (i.e., *Q* in this paper), and τ is normalized to 1 for ease of computation.

## 4 Model analysis

### 4.1 Non-government-subsidized model (NS)

In the benchmark model without government subsidies, both competing drug companies and healthcare institution engage in a Stackelberg differential game with the business objective of maximizing their own profits. Drug companies first determine the marginal profits of innovative and generic drugs *w*_1_ and *w*_2_. To expand the market demand, drug companies need to determine their respective R&D investment *r*_1_(*t*) and *r*_2_(*t*). Among them, innovative drug companies consider the impact of the decision on the future long-term benefits, while generic drug companies focus solely on consider the impact of their decision on immediate benefits. Healthcare institution provide medical services to patients and determine the marginal profit *p*. Healthcare institution need to improve the quality of medical services to attract patients to medical treatment, and in this way determine their own service efforts as *s*_1_(*t*), *s*_2_(*t*). Drug companies and healthcare institution aim to maximize their respective profits through the provision of products and services, and in the absence of government subsidies, the optimization problem is as follows.


(6) $$\begin{array}{l} \underset{r_{1}}{\max}\pi_{M_{1}}^{\text{NS}}(r_{1};r_{2};s_{1};s_{2})=\int_{0}^{+\infty }e^{-\rho t}\left\{w_{1}Q_{1}\left(t\right)-\frac{1}{2}r_{1}^{2}\left(t\right)\right\}\text{d}t\\ \underset{r_{2}}{\max}\pi_{M_{2}}^{\text{NS}}(r_{1};r_{2};s_{1};s_{2})=w_{2}Q_{2}\left(t\right)-\frac{1}{2}r_{2}^{2}\left(t\right)\\ \underset{s}{\max}\pi_{H}^{\text{NS}}(r_{1};r_{2};s_{1};s_{2})=\int_{0}^{+\infty }e^{-\rho t}\{p\left[Q_{1}\left(t\right)+Q_{2}\left(t\right)\right]-\frac{1}{2}s_{1}^{2}\left(t\right)\\ -\frac{1}{2}s_{2}^{2}\left(t\right)\}\text{d}t\\ \text{s}.\text{t. }\{\begin{array}{l} {\dot{G}}_{i}(t)=\mu r_{i}(t)-kG_{i}(t)\\ {\dot{G}}_{i}(0)=G_{0},\;i=1,2 \end{array} \end{array}$$

**Proposition 1:** The optimal performance indicators of the supply chain under the NS subsidy model are as follows:

(1) The R&D and innovation investments of the two competing manufacturers are:


$$\{\begin{array}{l} r_{1}^{\text{NS}}=w_{1}\left[\left(\alpha +d\right)+\frac{\mu \lambda }{\rho +k}\right]\\ \;r_{2}^{\text{NS}}=w_{2}\left(\alpha +d\right) \end{array};$$

(2) The service efforts of healthcare institution are:$s_{1}^{\text{NS}}=p\beta ,\;s_{2}^{\text{NS}}=p\beta$;

(3) The evolutionary paths of drug quality for each of the two competing manufacturers:


$$\{\begin{array}{l} G_{1}^{\text{NS}}(t)=G_{0}e^{-\rho t}+\left(1-e^{-\rho t}\right)\frac{\mu w_{1}}{k}\left[\left(\alpha +d\right)+\frac{\mu \lambda }{\rho +k}\right]\\ G_{2}^{\text{NS}}(t)=G_{0}e^{-\rho t}+\left(1-e^{-\rho t}\right)\frac{\mu w_{2}}{k}\left(\alpha +d\right) \end{array};$$

(4) The profits of the two competing drug companies and healthcare institution are:


$$\{\begin{array}{l} V_{{\text{M}}_{1}}^{\text{NS}}=\frac{w_{1}\lambda }{\rho +k}G_{1}+l_{1}\\ V_{{\text{M}}_{2}}^{\text{NS}}=\frac{w_{2}\lambda }{\rho }G_{2}+l_{2}\\ V_{\text{H}}^{\text{NS}}=\frac{p\lambda }{\rho +k}G_{1}+\frac{p\lambda }{\rho +k}G_{2}+l_{3} \end{array};$$

where,


$$\begin{array}{l} l_{1}=\frac{w_{1}}{\rho }\left[\varepsilon a-bw_{1}+p\beta^{2}-\left(\alpha +d\right)dw_{2}\right]\\ +\frac{1}{2\rho }{\left[w_{1}\left(\alpha +d\right)+\mu \frac{w_{1}\lambda }{\rho +k}\right]}^{2}\\ l_{2}=\frac{w_{2}}{\rho }\left[\left(1-\varepsilon \right)a-bw_{2}+p\beta^{2}-\left(\alpha +d\right)dw_{1}-\frac{d\mu w_{1}\lambda }{\rho +k}\right]\\ +\frac{1}{2\rho }w_{2}^{2}{\left(\alpha +d\right)}^{2}\\ l_{3}=\frac{1}{\rho }\left\{\begin{array}{l} p\left[a-\left(w_{1}+w_{2}\right)b+\left(w_{1}+w_{2}\right)\alpha \left(\alpha +d\right)\right]+p^{2}\beta^{2}\\ +\mu \frac{w_{1}\lambda }{\rho +k}\left(p\alpha +\mu \frac{p\lambda }{\rho +k}\right)+\left(\alpha +d\right)\left(w_{1}+w_{2}\right)\mu \frac{p\lambda }{\rho +k} \end{array}\right\} \end{array}$$

(5) Social welfare for:


$$SW^{NS}=\begin{array}{l} \frac{\left(w_{1}+p\right)\lambda }{\rho +k}G_{1}+\frac{1}{2}{\left[\varepsilon a-bw_{1}+\alpha w_{1}\left(\alpha +d\right)+\frac{\alpha w_{1}\mu \lambda }{\rho +k}+p\beta^{2}+\lambda G_{1}+d\left(w_{1}-w_{2}\right)\left(\alpha +d\right)+\frac{dw_{1}\mu \lambda }{\rho +k}\right]}^{2}\\ \begin{array}{l} \langle +\frac{1}{\rho }\left\{\begin{array}{l} w_{1}\left[\varepsilon a-bw_{1}+p\beta^{2}-\left(\alpha +d\right)dw_{2}\right]+w_{2}\left[\left(1-\varepsilon \right)a-bw_{2}+p\beta^{2}-\left(\alpha +d\right)dw_{1}-\frac{d\mu w_{1}\lambda }{\rho +k}\right]\\ +\frac{1}{2}{\left[w_{1}\left(\alpha +d\right)+\mu \frac{w_{1}\lambda }{\rho +k}\right]}^{2}+\frac{1}{2}w_{2}^{2}{\left(\alpha +d\right)}^{2}\\ p\left[a-\left(w_{1}+w_{2}\right)b+\left(w_{1}+w_{2}\right)\alpha \left(\alpha +d\right)\right]+p^{2}\beta^{2}\\ +\mu \frac{w_{1}\lambda }{\rho +k}\left(p\alpha +\mu \frac{p\lambda }{\rho +k}\right)+\left(\alpha +d\right)\left(w_{1}+w_{2}\right)\mu \frac{p\lambda }{\rho +k} \end{array}\right\}\rangle \\ +\frac{\left(w_{2}+p\right)\lambda }{\rho }G_{2}+\frac{1}{2}{\left[\left(1-\varepsilon \right)a-bw_{2}+\alpha w_{2}\left(\alpha +d\right)+p\beta^{2}+\lambda G_{2}+d\left(w_{2}-w_{1}\right)\left(\alpha +d\right)-\frac{dw_{1}\mu \lambda }{\rho +k}\right]}^{2} \end{array}\\ \; \end{array}.$$

For proof, see Appendix A.

Proposition 1 indicates that: (1) The increase in drug R&D investment and drug quality of the two competing drug companies is positively correlated with the increase in competitive intensity and marginal profit of the drug. Additionally, innovative drug company M_1_ is positively correlated with both the impact of R&D investment on drug quality and the effect of drug quality on demand as R&D investment and drug quality improve. (2) The service effort of a healthcare institution is positively correlated with both the increase in its marginal profit per unit of service and the factor of the effect of service effort on demand. (3) The overall profits of the two competing drug companies and the healthcare institution are positively correlated with drug quality, and the increase in marginal profit, the factor of the impact of the service effort on the demand, as well as the investment in drug R&D and innovation, the factor of the impact of the investment in drug R&D on the quality of the drug and the factor of the impact of the quality of the drug on the demand.

As the intensity of competition among drug companies increases, the gap between drug technology continues to widen, and the substitutability of the two in the market is gradually reduced. At this point, drug companies are choosing to increase drug R&D and innovation, in order to produce higher-quality and more effective innovative and generic drugs, aiming to gain better reputations and broader market demand.

### 4.2 Innovative drug subsidy model (IS)

In the IS subsidy model, the government provides a subsidy of θ proportion of R&D investment to innovative drug companies. As a condition, these companies need to offer a certain percentage of price discounts during the centralized drug procurement process, at which time the demand function for drugs becomes:


(7) $$\{\begin{array}{l} Q_{1}(t)=\varepsilon a-b\theta w_{1}+\alpha r_{1}\left(t\right)+\beta s_{1}\left(t\right)+\lambda {\dot{G}}_{1}\left(t\right)\\ +d\left(r_{1}\left(t\right)-r_{2}\left(t\right)\right)\\ Q_{2}(t)=\left(1-\varepsilon \right)a-bw_{2}+\alpha r_{2}\left(t\right)+\beta s_{2}\left(t\right)+\lambda G_{2}\left(t\right)\\ +d\left(r_{2}\left(t\right)-r_{1}\left(t\right)\right) \end{array}$$

The government provides R&D subsidies to: on the one hand, address the issue of insufficient investment in drug R&D activities by innovative drug companies, reduce the R&D risks of drug companies by sharing part of the R&D costs, encourage the production of high-quality domestically produced innovative drugs for the market, and foster the development of domestic innovative drug brands; on the other hand, promote the structural reform of the drug industry's drug categories, enabling domestically produced innovative drugs with the same efficacy but lower prices to gradually replace expensive imported drugs, thereby reducing patients' medical costs. The optimization issues are as follows:


(8) $$\begin{array}{l} \underset{r_{1}}{\max}\pi_{{\text{M}}_{1}}^{\text{IS}}(r_{1};r_{2};s_{1};s_{2})=\int_{0}^{+\infty }e^{-\rho t}\left\{w_{1}Q_{1}\left(t\right)-\frac{1}{2}\left(1-\theta \right)r_{1}^{2}\left(t\right)\right\}\text{d}t\\ \underset{r_{2}}{\max}\pi_{{\text{M}}_{2}}^{\text{IS}}(r_{1};r_{2};s_{1};s_{2})=w_{2}Q_{2}\left(t\right)-\frac{1}{2}r_{2}^{2}\left(t\right)\\ \underset{s}{\max}\pi_{\text{H}}^{\text{IS}}(r_{1};r_{2};s_{1};s_{2})=\int_{0}^{+\infty }e^{-\rho t}\{p\left[Q_{1}\left(t\right)+Q_{2}\left(t\right)\right]\\ -\frac{1}{2}s_{1}^{2}\left(t\right)-\frac{1}{2}s_{2}^{2}\left(t\right)\}\text{d}t\\ \text{s.t. }\{\begin{array}{l} {\dot{G}}_{i}(t)=\mu r_{i}(t)-k{\dot{G}}_{i}(t)\\ {\dot{G}}_{i}(0)=G_{0},\;i=1,2 \end{array} \end{array}$$

**Proposition 2:** The optimal performance indicators of the supply chain under the IS subsidy model are as follows:

(1) The R&D innovation inputs of the two competing manufacturers are:


$$\{\begin{array}{l} r_{1}^{\text{IS}}=\frac{w_{1}}{\left(1-\theta \right)}\left[\left(\alpha +d\right)+\frac{\mu \lambda }{k+\rho }\right]\\ r_{2}^{\text{IS}}=w_{2}\left(\alpha +d\right) \end{array}$$

(2) The service efforts of healthcare institution are: $s_{1}^{\text{IS}}=p\beta ,\;s_{2}^{\text{IS}}=p\beta$;

(3) The evolutionary paths of drug quality for each of the two competing manufacturers:


$$\{\begin{array}{l} G_{1}^{\text{IS}}(t)=G_{0}e^{-\rho t}+\left(1-e^{-\rho t}\right)\frac{\mu w_{1}}{k\left(1-\theta \right)}\left[\left(\alpha +d\right)+\frac{\mu \lambda }{\rho +k}\right]\\ G_{2}^{\text{IS}}(t)=G_{0}e^{-\rho t}+\left(1-e^{-\rho t}\right)\frac{\mu w_{2}}{k}\left(\alpha +d\right) \end{array};$$

(4) The profits of the two competing manufacturers and healthcare institution are:


$$\{\begin{array}{l} V_{{\text{M}}_{1}}^{\text{IS}}=\frac{w_{1}\lambda }{k+\rho }G_{1}+l_{4}\\ V_{{\text{M}}_{2}}^{\text{IS}}=\frac{w_{2}\lambda }{\rho }G_{2}+l_{5}\\ V_{\text{H}}^{\text{IS}}=\frac{p\lambda }{\rho +k}G_{1}+\frac{p\lambda }{\rho +k}G_{2}+l_{6} \end{array};$$

where,


$$\begin{array}{l} l_{4}=\frac{w_{1}}{\rho }\left[\varepsilon a-bw_{1}+p\beta^{2}-dw_{2}\left(\alpha +d\right)\right]\\ +\frac{1}{2\rho \left(1-\theta \right)}{\left[w_{1}\left(\alpha +d\right)+\mu \frac{w_{1}\lambda }{k+\rho }\right]}^{2}\\ l_{5}=\frac{w_{2}}{\rho }\left[\left(1-\varepsilon \right)a-bw_{2}+p\beta^{2}-\frac{w_{1}d}{\left(1-\theta \right)}\left(\alpha +d\right)-\frac{\mu dw_{1}\lambda }{\left(1-\theta \right)\left(\rho +k\right)}\right]\\ +\frac{1}{2\rho }w_{2}2{\left(\alpha +d\right)}^{2}\\ l_{6}=\frac{1}{\rho }\left\{\begin{array}{l} p\left[a-b\left(w_{1}+w_{2}\right)+\alpha \left(\alpha +d\right)\left(\frac{w_{1}}{1-\theta }+w_{2}\right)\right]+p^{2}\beta^{2}\\ +\frac{\mu w_{1}\lambda }{\left(\rho +k\right)\left(1-\theta \right)}\left(p\alpha +\mu \frac{p\lambda }{\rho +k}\right)+\left(\alpha +d\right)\frac{\mu p\lambda }{\rho +k}\left(\frac{w_{1}}{1-\theta }+w_{2}\right) \end{array}\right\} \end{array}$$

(5) Social welfare for:


$$SW^{\text{IS}}=\begin{array}{l} \;\frac{1}{2}\left[\varepsilon a-b\theta w_{1}+\frac{\alpha w_{1}\left(\alpha +d\right)}{\left(1-\theta \right)}+\frac{\mu \lambda \alpha w_{1}}{\left(1-\theta \right)\left(k+\rho \right)}+p\beta^{2}+\lambda G_{1}\left(t\right)+d\left(\frac{w_{1}}{1-\theta }-w_{2}\right)\left(\alpha +d\right)+\frac{dw_{1}\mu \lambda }{\left(1-\theta \right)\left(k+\rho \right)}\right]\\ \begin{array}{l} \langle +\frac{1}{2\rho }\left\{\begin{array}{l} w_{1}\left[\varepsilon a-bw_{1}+p\beta^{2}-dw_{2}\left(\alpha +d\right)\right]+\frac{1}{2\left(1-\theta \right)}{\left[w_{1}\left(\alpha +d\right)+\mu \frac{w_{1}\lambda }{k+\rho }\right]}^{2}\\ +w_{2}\left[\left(1-\varepsilon \right)a-bw_{2}+p\beta^{2}-\frac{w_{1}d}{\left(1-\theta \right)}\left(\alpha +d\right)-\frac{\mu dw_{1}\lambda }{\left(1-\theta \right)\left(\rho +k\right)}\right]+\frac{1}{2}w_{2}2{\left(\alpha +d\right)}^{2}\\ +p\left[a-b\left(w_{1}+w_{2}\right)+\alpha \left(\alpha +d\right)\left(\frac{w_{1}}{1-\theta }+w_{2}\right)\right]\\ +p^{2}\beta^{2}+\frac{\mu w_{1}\lambda }{\left(\rho +k\right)\left(1-\theta \right)}\left(p\alpha +\mu \frac{p\lambda }{\rho +k}\right)+\left(\alpha +d\right)\frac{\mu p\lambda }{\rho +k}\left(\frac{w_{1}}{1-\theta }+w_{2}\right) \end{array}\right\}+\frac{\left(w_{1}+p\right)\lambda }{k+\rho }G_{1}\;\rangle \\ \;+\frac{1}{2}\left[\left(1-\varepsilon \right)a-bw_{2}+\alpha w_{2}\left(\alpha +d\right)+p\beta^{2}+\lambda G_{2}\left(t\right)+d\left(w_{2}-\frac{w_{1}}{1-\theta }\right)\left(\alpha +d\right)-\frac{dw_{1}\mu \lambda }{\left(1-\theta \right)\left(k+\rho \right)}\right]+\frac{\left(w_{2}+p\right)\lambda }{\rho }G_{2} \end{array}\\ \; \end{array}.$$

For proof, see Appendix A.

Proposition 2 illustrates that: (1) Both drug companies' investment in drug R&D and improvement in drug quality are directly related to the increase in competitive intensity and the marginal profit of drugs. Additionally, innovative drug company M_1_ is positively correlated with the proportion of government subsidies, the factor of R&D investment on drug quality, and the factor of drug quality on demand, as R&D investment and drug quality improve. (2) The profits of both drug companies and the healthcare institution are positively correlated with the drug quality and the marginal profit of the drugs. The profits of generic drug companies M_2_ are negatively correlated with the proportion of government subsidies, while the profits of innovative drug company M_1_ and the healthcare institution are positively correlated with the proportion of government subsidies. (3) The service effort of healthcare institution is positively correlated with an increase in its marginal profit per unit of service, as well as with the factor of the impact of service effort on demand.

Government subsidies have stimulated R&D investment by innovative drug companies, leading to improvements in the quality of innovative drugs and overall corporate profits. However, subsidies have widened the technological gap between the two competing drug research and development systems, intensifying market competition. This has forced generic drug companies to lose market share and has reduced their profit levels. Therefore, when the government provides subsidies to innovative drug companies, it also needs to consider the revenue problems of generic drug companies, and ensure their survival of generic drug companies on the basis of effective subsidies. As the market demand for innovative drugs expands, the demand for healthcare institutions' services for the corresponding drugs also increases, allowing healthcare institutions to earn greater profits from their services. However, due to cost factors and the influence of the “zero-differential rate” policy, the service efforts of the healthcare institution have remained unchanged.

### 4.3 Generic drug subsidy model (GS)

In the GS subsidy model, the government provides R&D investment subsidies to generic drug companies in proportion δ. The purpose is to improve the quality of generic drugs, enable them to enter the market as soon as possible to replace the original drugs, reduce drug prices, and reduce health expenditures. Simultaneously, these subsidies can promote the industrial transformation and upgrading of the generic drug companies, encourage the establishment of a long-term development strategy, and consider the impact of current decisions on future long-term interests. The optimization problem is as follows:


(9) $$\begin{array}{l} \underset{r_{1}}{\max}\pi_{{\text{M}}_{1}}^{\text{GS}}(r_{1};r_{2};s_{1};s_{2})=\int_{0}^{+\infty }e^{-\rho t}\left\{w_{1}Q_{1}\left(t\right)-\frac{1}{2}r_{1}^{2}\left(t\right)\right\}\text{d}t\\ \underset{r_{2}}{\max}\pi_{{\text{M}}_{2}}^{\text{GS}}(r_{1};r_{2};s_{1};s_{2})=\int_{0}^{+\infty }e^{-\rho t}\left\{w_{2}Q_{2}\left(t\right)-\frac{1}{2}\left(1-\delta \right)r_{2}^{2}\left(t\right)\right\}\text{d}t\\ \underset{s}{\max}\pi_{\text{H}}^{\text{GS}}(r_{1};r_{2};s_{1};s_{2})=\int_{0}^{+\infty }e^{-\rho t}\{p\left[Q_{1}\left(t\right)+Q_{2}\left(t\right)\right]\\ -\frac{1}{2}s_{1}^{2}\left(t\right)-\frac{1}{2}s_{2}^{2}\left(t\right)\}\text{d}t\\ \text{s.t. }\{\begin{array}{l} {\dot{G}}_{1}(t)=\mu r_{1}(t)-kG_{1}(t),G_{1}\left(0\right)=G_{0}\\ {\dot{G}}_{2}(t)=\mu r_{2}(t)-kG_{2}(t),G_{2}\left(0\right)=G_{0} \end{array} \end{array}$$

**Proposition 3:** The optimal performance indicators of the supply chain under the GS subsidy model are as follows:

(1) The R&D innovation inputs of the two competing manufacturers are:


$$\{\begin{array}{l} r_{1}^{\text{GS}}=w_{1}\left[\left(\alpha +d\right)+\mu \frac{\lambda }{\rho +k}\right]\\ r_{2}^{\text{GS}}=\frac{w_{2}}{\left(1-\delta \right)}\left[\left(\alpha +d\right)+\mu \frac{\lambda }{\rho +k}\right] \end{array};$$

(2) The service efforts of healthcare institution are:$s_{1}^{\text{GS}}=p\beta ,\;s_{2}^{\text{GS}}=p\beta$;

(3) The evolutionary paths of drug quality for each of the two competing manufacturers:


$$\{\begin{array}{l} G_{1}^{\text{GS}}(t)=G_{0}e^{-\rho t}+\left(1-e^{-\rho t}\right)\frac{\mu w_{1}}{k}\left[\left(\alpha +d\right)+\frac{\mu \lambda }{\rho +k}\right]\\ G_{2}^{\text{GS}}(t)=G_{0}e^{-\rho t}+\left(1-e^{-\rho t}\right)\frac{\mu w_{2}}{k\left(1-\delta \right)}\left[\left(\alpha +d\right)+\mu \frac{\lambda }{\rho +k}\right] \end{array};$$

(4) The profits of the two competing manufacturers and healthcare institution are:


$$\{\begin{array}{l} V_{{\text{M}}_{1}}^{\text{GS}}=\frac{w_{1}\lambda }{\rho +k}G_{1}+l_{7}\\ V_{{\text{M}}_{2}}^{\text{GS}}=\frac{w_{2}\lambda }{\rho +k}G_{2}+l_{8}\\ V_{\text{H}}^{\text{GS}}=\frac{\lambda p}{\rho +k}G_{1}+\frac{\lambda p}{\rho +k}G_{2}+l_{9} \end{array};$$

where,


$$\begin{array}{l} l_{7}=\frac{w_{1}}{\rho }\left[\varepsilon a-bw_{1}+p\beta^{2}-d\left(\alpha +d\right)\frac{w_{2}}{1-\delta }-\frac{\mu dw_{2}\lambda }{\left(\rho +k\right)\left(1-\delta \right)}\right]\\ +\frac{1}{2\rho }{\left[w_{1}\left(\alpha +d\right)+\mu \frac{w_{1}\lambda }{\rho +k}\right]}^{2}\\ l_{8}=\frac{w_{2}}{\rho }\left[\left(1-\varepsilon \right)a-bw_{2}+p\beta^{2}-d\left(\alpha +d\right)w_{1}-\frac{\mu dw_{1}\lambda }{\rho +k}\right]\\ +\frac{1}{2\left(1-\delta \right)\rho }{\left[w_{2}\left(\alpha +d\right)+\mu \frac{w_{2}\lambda }{\rho +k}\right]}^{2}\\ l_{9}=\frac{1}{\rho }\left\{\begin{array}{l} p\left[a-b\left(w_{1}+w_{2}\right)+\alpha \left(w_{1}+\frac{w_{2}}{1-\delta }\right)\left(\alpha +d+\frac{\mu \lambda }{\rho +k}\right)\right]\\ +p^{2}\beta^{2}+\frac{\mu \lambda p}{\rho +k}[\left(w_{1}+\frac{w_{2}}{1-\delta }\right)\left(\alpha +d\right)+\frac{\mu \lambda }{\rho +k}(\mu w_{1}\\ +\frac{w_{2}}{1-\delta })] \end{array}\right\} \end{array}$$

(5) Social welfare for:


$$\begin{array}{l} \;\frac{1}{2}{\left[\varepsilon a-bw_{1}+\alpha w_{1}\left(\alpha +d\right)+\frac{\alpha w_{1}\mu \lambda }{\rho +k}+\beta^{2}p+\lambda G_{1}\left(t\right)+d\left(w_{1}-\frac{w_{2}}{1-\delta }\right)\left(\alpha +d\right)+\frac{d\mu \lambda }{\rho +k}\left(w_{1}+\frac{w_{2}}{1-\delta }\right)\right]}^{2}+\frac{\left(w_{2}+p\right)\lambda }{\rho +k}G_{2}\\ SW^{GS}=\langle \frac{\left(w_{1}+p\right)\lambda }{\rho +k}G_{1}+\frac{1}{\rho }\left\{\begin{array}{l} w_{1}\left[\varepsilon a-bw_{1}+p\beta^{2}-d\left(\alpha +d\right)\frac{w_{2}}{1-\delta }-\frac{\mu dw_{2}\lambda }{\left(\rho +k\right)\left(1-\delta \right)}\right]+\frac{1}{2}{\left[w_{1}\left(\alpha +d\right)+\mu \frac{w_{1}\lambda }{\rho +k}\right]}^{2}\\ +w_{2}\left[\left(1-\varepsilon \right)a-bw_{2}+p\beta^{2}-d\left(\alpha +d\right)w_{1}-\frac{\mu dw_{1}\lambda }{\rho +k}\right]+\frac{1}{2\left(1-\delta \right)}{\left[w_{2}\left(\alpha +d\right)+\mu \frac{w_{2}\lambda }{\rho +k}\right]}^{2}\\ p\left[a-b\left(w_{1}+w_{2}\right)+\alpha \left(w_{1}+\frac{w_{2}}{1-\delta }\right)\left(\alpha +d+\frac{\mu \lambda }{\rho +k}\right)\right]+p^{2}\beta^{2}\\ +\frac{\mu \lambda p}{\rho +k}\left[\left(w_{1}+\frac{w_{2}}{1-\delta }\right)\left(\alpha +d\right)+\frac{\mu \lambda }{\rho +k}\left(\mu w_{1}+\frac{w_{2}}{1-\delta }\right)\right] \end{array}\right\}\;\rangle \\ \;+\frac{1}{2}{\left[\left(1-\varepsilon \right)a-bw_{2}+\frac{\alpha w_{2}}{1-\delta }\left(\alpha +d\right)+\frac{\alpha w_{2}\mu \lambda }{\left(1-\delta \right)\left(\rho +k\right)}+\beta^{2}p+\lambda G_{2}\left(t\right)+d\left(\frac{w_{2}}{1-\delta }-w_{1}\right)\left(\alpha +d\right)+\frac{d\mu \lambda }{\rho +k}\left(\frac{w_{2}}{\left(1-\delta \right)}+w_{1}\right)\right]}^{2} \end{array}$$

For proof, see Appendix A.

Proposition 3 shows that: (1) There is a direct relationship between the improvement in drug quality and the drug R&D investment of the two drug companies, the intensity of competition and the marginal profit of drugs. In addition, the factors influencing R&D investment on drug quality and the influence factor of drug quality on demand also positively contribute to the improvement of R&D investment and drug quality. Generic drug companies M_2_ receives government subsidies for R&D investment, thus promoting the improvement of drug quality. (2) The service effort of a healthcare institution is positively correlated with the increase in its marginal profit per unit of service, as well as with the factor of the impact of service effort on demand. (3) The profits of the two drug companies and healthcare institution are positively correlated with drug quality and the marginal profit of the drug. The profit of generic drug company M_2_ is positively related to the proportion of government subsidies, whereas the profit of innovative drug company M_1_ is negatively correlated with this proportion.

Government subsidies for generic drugs have stimulated R&D and innovation investment by generic drug companies, improved drug quality and profitability, and provided a sustainable development direction for generic drug companies in the competitive market. On the one hand, companies can technologically upgrade the existing generic drug production process, improve drug quality, enhance market competitiveness, and reduce drug costs for patients; on the other hand, after receiving sufficient subsidy funds, companies will engage in innovative R&D on the existing synthesis process and pharmacology, and the main body of the industry will be transformed from generic drug production to the R&D of innovative drugs. Both paths are conducive to the healthy development of China's drug industry and address the survival problem faced by some generic drug companies. And subsidies lead to increased market competition, although the loss of some of the profits of innovative drug companies, but this competition is beneficial to the social environment of medical care. Patients have more opportunities to choose low-priced, high-quality drugs, further reducing their medical burden and addressing the issue of “expensive medical care”.

### 4.4 Healthcare institution subsidy model (HS)

In the HS subsidy model, the government encourages healthcare institution at all levels to prioritize the use of domestically produced generic drugs that have passed consistency evaluation, in order to support the scale development of domestically produced generic drugs and reduce overall medical costs of the society. The government encourages healthcare institution at all levels to prioritize the use of domestically produced generic drugs that have passed consistency evaluation, and a subsidy incentive η, based on a proportion of the centralized purchasing price, is provided to healthcare institutions that meet the required standards for the amounts of generic drugs used. At the same time, generic companies need to maintain the long-term market competitiveness of their drugs to prevent them from being removed from the centralized purchasing catalog. Therefore, they are compelled to consider the impact of their decisions on future development. The optimization problem is as follows:


(10) $$\begin{array}{l} \underset{r_{1}}{\max}\pi_{{\text{M}}_{1}}^{\text{HS}}(r_{1};r_{2};s_{1};s_{2})=\int_{0}^{+\infty }e^{-\rho t}\left\{w_{1}Q_{1}\left(t\right)-\frac{1}{2}r_{1}^{2}\left(t\right)\right\}\text{d}t\\ \underset{r_{2}}{\max}\pi_{{\text{M}}_{2}}^{\text{HS}}(r_{1};r_{2};s_{1};s_{2})=\int_{0}^{+\infty }e^{-\rho t}\left\{w_{2}Q_{2}\left(t\right)-\frac{1}{2}r_{2}^{2}\left(t\right)\right\}\text{d}t\\ \underset{s}{\max}\pi_{\text{H}}^{\text{HS}}(r_{1};r_{2};s_{1};s_{2})=\int_{0}^{+\infty }e^{-\rho t}\{pQ_{1}\left(t\right)+(p+\eta w_{2})Q_{2}\left(t\right)\\ -\frac{1}{2}s_{1}^{2}\left(t\right)-\frac{1}{2}s_{2}^{2}\left(t\right)\}\text{d}t\\ \text{s.t. }\{\begin{array}{l} {\dot{G}}_{1}(t)=\mu r_{1}(t)-kG_{1}(t),G_{1}\left(0\right)=G_{0}\\ {\dot{G}}_{2}(t)=\mu r_{2}(t)-kG_{2}(t),G_{2}\left(0\right)=G_{0} \end{array} \end{array}$$

**Proposition 4:** The optimal performance indicators of the supply chain under the HS subsidy model are as follows:

(1) The R&D innovation inputs of the two competing manufacturers are:


$$\{\begin{array}{l} r_{1}^{\text{HS}}=w_{1}\left[\left(\alpha +d\right)+\mu \frac{\lambda }{\rho +k}\right],\\ \;r_{2}^{\text{HS}}=w_{2}\left[\left(\alpha +d\right)+\mu \frac{\lambda }{\rho +k}\right] \end{array};$$

(2) The service efforts of healthcare institution are:

(3) The evolutionary paths of drug quality for each of the two competing manufacturers:


$$\{\begin{array}{l} G_{1}^{\text{HS}}(t)=G_{0}e^{-\rho t}+\left(1-e^{-\rho t}\right)\frac{\mu w_{1}}{k}\left[\left(\alpha +d\right)+\frac{\mu \lambda }{\rho +k}\right]\\ G_{2}^{\text{HS}}(t)=G_{0}e^{-\rho t}+\left(1-e^{-\rho t}\right)\frac{\mu w_{2}}{k}\left[\left(\alpha +d\right)+\mu \frac{\lambda }{\rho +k}\right] \end{array};$$

(4) The profits of the two competing manufacturers and healthcare institution are:


$$\{\begin{array}{l} V_{{\text{M}}_{1}}^{\text{HS}}=\frac{w_{1}\lambda }{\rho +k}G_{1}+l_{10}\\ V_{{\text{M}}_{2}}^{\text{GS}}=\frac{w_{2}\lambda }{\rho +k}G_{2}+l_{11}\\ V_{\text{H}}^{\text{GS}}=\frac{\lambda p}{\rho +k}G_{1}+\frac{\lambda \left(p+\eta w_{2}\right)}{\rho +k}G_{2}+l_{12} \end{array};$$

where,


$$\begin{array}{l} l_{10}=\frac{w_{1}}{\rho }\left[\varepsilon a-bw_{1}+p\beta^{2}-d\left(\alpha +d\right)w_{2}-\frac{\mu dw_{2}\lambda }{\rho +k}\right]\\ +\frac{1}{2\rho }{\left[w_{1}\left(\alpha +d\right)+\mu \frac{w_{1}\lambda }{\rho +k}\right]}^{2}\\ l_{11}=\frac{w_{2}}{\rho }\left[\left(1-\varepsilon \right)a-bw_{2}+\left(p+\eta w_{2}\right)\beta^{2}-d\left(\alpha +d\right)w_{1}-\frac{\mu dw_{1}\lambda }{\rho +k}\right]\\ +\frac{1}{2\rho }{\left[w_{2}\left(\alpha +d\right)+\mu \frac{w_{2}\lambda }{\rho +k}\right]}^{2}\\ l_{12}=\frac{1}{\rho }\left\{\begin{array}{l} p\left[a+\left(\alpha^{2}+\alpha d-b+\frac{\lambda \alpha \mu }{\rho +k}\right)\left(w_{1}+w_{2}\right)\right]\\ +\beta^{2}\left(p^{2}+p\eta w_{2}+\frac{1}{2}\eta^{2}w_{2}2\right)\\ +\eta w_{2}[\left(1-\varepsilon \right)a-bw_{2}+w_{2}{\left(\alpha +d\right)}^{2}\\ +\mu \frac{w_{2}\lambda }{\rho +k}d\left(\alpha +d\right)w_{1}+\frac{\lambda \mu d}{\rho +k}\left(w_{2}-w_{1}\right)]\\ \frac{\lambda \mu }{\rho +k}\left[pw_{1}+\left(p+\eta w_{2}\right)w_{2}\right]{\left(\alpha +d+\mu \frac{\lambda }{\rho +k}\right)}^{2} \end{array}\right\} \end{array}$$

(5) Social welfare for:


$$\begin{array}{l} \;\frac{1}{2}{\left[\varepsilon a-bw_{1}+\alpha w_{1}\left(\alpha +d\right)+\frac{\alpha w_{1}\mu \lambda }{\rho +k}+p\beta^{2}+\lambda G_{1}\left(t\right)+d\left(w_{1}-w_{2}\right)\left(\alpha +d+\frac{\mu \lambda }{\rho +k}\right)\right]}^{2}+\frac{\lambda \left(w_{2}+p+\eta w_{2}\right)}{\rho +k}G_{2}\\ SW^{\text{HS}}=\langle +\frac{1}{\rho }\left\{\begin{array}{l} w_{1}\left[\varepsilon a-bw_{1}+p\beta^{2}-d\left(\alpha +d\right)w_{2}-\frac{\mu dw_{2}\lambda }{\rho +k}\right]+\frac{1}{2}{\left[w_{1}\left(\alpha +d\right)+\mu \frac{w_{1}\lambda }{\rho +k}\right]}^{2}\\ +w_{2}\left[\left(1-\varepsilon \right)a-bw_{2}+\left(p+\eta w_{2}\right)\beta^{2}-d\left(\alpha +d\right)w_{1}-\frac{\mu dw_{1}\lambda }{\rho +k}\right]+\frac{1}{2}{\left[w_{2}\left(\alpha +d\right)+\mu \frac{w_{2}\lambda }{\rho +k}\right]}^{2}\\ +p\left[a+\left(\alpha^{2}+\alpha d-b+\frac{\lambda \alpha \mu }{\rho +k}\right)\left(w_{1}+w_{2}\right)\right]+\beta^{2}\left(p^{2}+p\eta w_{2}+\frac{1}{2}\eta^{2}w_{2}2\right)\\ +\eta w_{2}\left[\left(1-\varepsilon \right)a-bw_{2}+w_{2}{\left(\alpha +d\right)}^{2}+\mu \frac{w_{2}\lambda }{\rho +k}d\left(\alpha +d\right)w_{1}+\frac{\lambda \mu d}{\rho +k}\left(w_{2}-w_{1}\right)\right]\\ +\frac{\lambda \mu }{\rho +k}\left[pw_{1}+\left(p+\eta w_{2}\right)w_{2}\right]{\left(\alpha +d+\mu \frac{\lambda }{\rho +k}\right)}^{2} \end{array}\right\}+\frac{\left(w_{1}+p\right)\lambda }{\rho +k}G\;\rangle \\ \;+\frac{1}{2}{\left[\left(1-\varepsilon \right)a-bw_{2}+\alpha w_{2}\left(\alpha +d\right)+\frac{\mu \alpha w_{2}\lambda }{\rho +k}+\left(p+\eta w_{2}\right)\beta^{2}+\lambda G_{2}\left(t\right)+d\left(w_{2}-w_{1}\right)\left(\alpha +d+\frac{\mu \lambda }{\rho +k}\right)\right]}^{2}1 \end{array}$$

For proof, see Appendix A.

Proposition 4 shows that: (1) The drug R&D investment and quality improvement of the two competing drug companies are directly related to the intensity of competition and the marginal profits of the drugs. In addition, the factor of R&D investment on drug quality and the factor of drug quality on demand also positively contribute to the improvement of R&D investment and drug quality. (2) The service effort of healthcare institution is closely related to the increase in their marginal profit per unit of service. Additionally, the factor of service effort on demand positively affects the service quality of healthcare institution. The service effort of healthcare institution is also positively related to the marginal profit of generic drugs and the proportion of government subsidies. (3) The profits of the two drug companies and healthcare institution are positively related to the quality of the drugs and the marginal profit of the drugs. The profit of innovative drug company M_1_ is negatively related to the proportion of government subsidies, while the profit of generic drug company M_2_ and healthcare institution is positively related to the proportion of government subsidies.

The government provides healthcare institution with assessment and incentive mechanisms to promote the use of generic drugs. To obtain more incentive funds, healthcare institution will alter their healthcare service strategies to guide patients toward prioritize the use of generics, ensuring a sufficient share of generic drugs in their sales. In addition, when healthcare institution changes their service strategies to increase the market demand for generic drugs, generic companies can generate more sales profits and are incentivized to make strategic plans for long-term growth. Meanwhile, without the support of government subsidies, innovative drug companies will not blindly alter their R&D decisions and the quality of their drugs will remain unchanged, considering the R&D costs and risk factors they are willing to accept.

## 5 Analysis of model results

This section builds on the previous section by first conducting a sensitivity analysis of the relevant parameters, followed by a comparison of the optimal decisions, profits, and social welfare of competing drug companies and healthcare providers under the four subsidy models, in order to draw more comprehensive conclusions and managerial insights.

**Corollary 1:** The effect of innovative drug subsidy ratio θ on optimal R&D investment, profit level and drug quality of competing drug companies:


$$\frac{\partial r_{1}^{IS}}{\partial \theta }>0,\frac{\partial r_{2}^{IS}}{\partial \theta }=0,\frac{\partial V_{1}^{IS}}{\partial \theta }>0,\frac{\partial V_{2}^{IS}}{\partial \theta }<0,\frac{\partial G_{1}^{IS}}{\partial \theta }>0,\frac{\partial G_{2}^{IS}}{\partial \theta }=0$$

Innovative drug companies' R&D investment *r*_1_, drug quality *G*_1_, and profit are positively correlated with subsidy ratio θ. Generic drug companies' profit *V*_2_ is negatively correlated with subsidy ratio θ. Government subsidies have effectively promoted the research and development of innovative drug products. The increase in the proportion of subsidies has encouraged innovative drug companies to invest more in R&D funds, and the quality of drugs has been improved. This has further widened the technological gap between drugs and increased the intensity of competition in the market. As the generic drug companies' R&D focuses solely on the impact of current benefits, and without change the R&D strategy according to the changes in the intensity of market competition in order to improve the image of the enterprise and maintain the competitiveness of generic drugs in the market, which forces generic drug companies to lose part of their market share, and further reducing their profit levels.

**Corollary 2:** The effect of generic subsidy ratio δ on optimal R&D investment, profit level and drug quality of competing drug companies:


$$\begin{array}{l} \frac{\partial r_{1}^{GS}}{\partial \delta }=0,\frac{\partial r_{2}^{GS}}{\partial \delta }>0,\frac{\partial V_{1}^{GS}}{\partial \delta }<0,\frac{\partial V_{2}^{GS}}{\partial \delta }>0,\frac{\partial G_{1}^{GS}}{\partial \delta }\\ \;=0,\frac{\partial G_{2}^{GS}}{\partial \delta }>0 \end{array}$$

Generic drug companies' R&D investment *r*_2_, drug quality, profit *V*_2_ and subsidy ratio δ are positively correlated; innovative drug companies' profit *V*_1_ and subsidy ratio δ are negatively correlated. Government subsidies effectively promote the R&D and innovation of generic drug companies, and improve drug quality while maintaining their price advantage. This reduces the medical burden on low- income patients and promotes the high-quality development of the overall healthcare sector. For innovative drug companies, the R&D investment required for innovative drugs is significantly higher than that for generic drugs. To reduce R&D risks, innovative drug companies are more willing to maintain the current decision-making, therefore they voluntarily sacrificing part of their market share. Generic drug companies can expand their market share through technological advancements and price advantages, significantly promoting the transformation and scaling of their operations.

**Corollary 3:** Impact of healthcare provider subsidy ratio η on profit levels and service effort of drug companies and healthcare providers:


$$\frac{\partial s_{1}^{GS}}{\partial \eta }=0,\frac{\partial s_{2}^{GS}}{\partial \eta }>0,\frac{\partial V_{1}^{GS}}{\partial \eta }<0,\frac{\partial V_{2}^{GS}}{\partial \eta }>0,\frac{\partial V_{H}^{GS}}{\partial \eta }>0$$

Effort *s*_2_ and profit *V*_H_ of generic drug services in healthcare institution and profit *V*_2_ of generic drug companies are positively correlated with subsidy ratio η; profit *V*_1_ of innovative drug companies is negatively correlated with subsidy ratio η. The early national drug negotiations, which stated that “original drugs do not account for a large proportion of drugs, while generic drugs do,” significantly hindered healthcare institutions' enthusiasm for using generic drugs. In some cases, healthcare institutions resorted to increasing examination costs or other drug services to meet the “drug proportion” targets and compensate for losses. At this point, government subsidies can effectively alleviate the difficulties of healthcare institution in drug usage. To obtain more subsidies, healthcare institution will change the medical service strategy to guide patients to give priority to the use of generic drugs, ensuring an enough generic drugs in the sales of drugs. The implementation of this subsidy policy reduces the operational burden of healthcare institution, promotes revenue reform of public healthcare institution, and reduces the cost of healthcare for patients.

**Corollary 4:** The magnitude of drug companies' investment in R&D and innovation, healthcare providers' service efforts, and drug quality under the four subsidy models are related as follows:


$$\begin{array}{l} r_{1}^{\text{IS}}>r_{1}^{\text{NS}}=r_{1}^{\text{GS}}=r_{1}^{\text{HS}},r_{2}^{\text{GS}}>r_{2}^{\text{HS}}>r_{2}^{\text{NS}}=r_{2}^{\text{IS}},\\ s_{1}^{\text{NS}}=s_{1}^{\text{IS}}=s_{1}^{\text{GS}}=s_{1}^{\text{HS}},s_{2}^{\text{HS}}>s_{2}^{\text{NS}}=s_{2}^{\text{IS}}=s_{2}^{\text{GS}},\\ \underset{t\to \infty }{\lim}G_{1}^{\text{IS}}>\underset{t\to \infty }{\lim}G_{1}^{\text{NS}}=\underset{t\to \infty }{\lim}G_{1}^{\text{GS}}=\underset{t\to \infty }{\lim}G_{1}^{\text{HS}},\\ \underset{t\to \infty }{\lim}G_{2}^{\text{GS}}>\underset{t\to \infty }{\lim}G_{2}^{\text{HS}}>\underset{t\to \infty }{\lim}G_{2}^{\text{NS}}=\underset{t\to \infty }{\lim}G_{2}^{\text{IS}} \end{array}$$

**Corollary 5:** The relationship between the size of the profits of the two competing drug companies and healthcare providers at steady state under the four subsidy models are:


(11) $$\begin{array}{l} \underset{t\to \infty }{\lim}V_{{\text{M}}_{1}}^{\text{IS}}>\underset{t\to \infty }{\lim}V_{{\text{M}}_{1}}^{\text{NS}}>\underset{t\to \infty }{\lim}V_{{\text{M}}_{1}}^{\text{HS}}>\underset{t\to \infty }{\lim}V_{{\text{M}}_{1}}^{\text{GS}},\\ \underset{t\to \infty }{\lim}V_{{\text{M}}_{2}}^{\text{GS}}>\underset{t\to \infty }{\lim}V_{{\text{M}}_{2}}^{\text{HS}}>\underset{t\to \infty }{\lim}V_{{\text{M}}_{2}}^{\text{NS}}>\underset{t\to \infty }{\lim}V_{\text{M}2}^{\text{IS}},\\ \underset{t\to \infty }{\lim}V_{\text{H}}^{\text{HS}}>\underset{t\to \infty }{\lim}V_{\text{H}}^{\text{IS}}>\underset{t\to \infty }{\lim}V_{\text{H}}^{\text{GS}}>\underset{t\to \infty }{\lim}V_{\text{H}}^{\text{NS}} \end{array}$$

Corollaries 4 and 5 show that R&D innovation investment, drug quality, and profit are maximized under the IS subsidy model for innovative drug companies and under the GS subsidy model for generic drug companies. Healthcare provider profit and generic service efforts are optimized under the HS subsidy model, while innovator service effort remain the same across all four models. When the government subsidizes generic companies, the impact of the subsidy on drug quality is greater than the impact on market demand. As a result, profits under the R&D subsidy strategy of generic companies exceed those under the revenue subsidy strategy, whereas the opposite holds true for healthcare providers under the HS subsidy model. The government's subsidy policy plays a crucial role in guiding the R&D of drug companies and healthcare institution. It facilitates the transformation of the drug supply structure, which not only boosts market demand for generic drugs and promotes the rapid development of innovative drugs, but also enhances patients' satisfaction with medical treatment and the overall social welfare.

## 6 Numerical analysis

In this section, the relevant parameters are further analyzed through numerical examples. Combined with the reality and referring to the assumptions of Ma et al. (50) and Wen and Liu (52), the basic parameters are set as follows:


$$\begin{array}{l} a=1;b=0.4;\varepsilon =0.3,\alpha =0.4;\beta =0.6,\lambda =0.4,\\ d=0.6,\mu =0.6,k=0.1,\rho =0.1,w_{1}=2,w_{2}=1,p=1,\theta =0.4,\\ \delta =0.4,\eta =0.2,U_{0}=0.1 \end{array}$$

### 6.1 Impact of competitive intensity on drug companies' R&D investment

**Figure 2:**
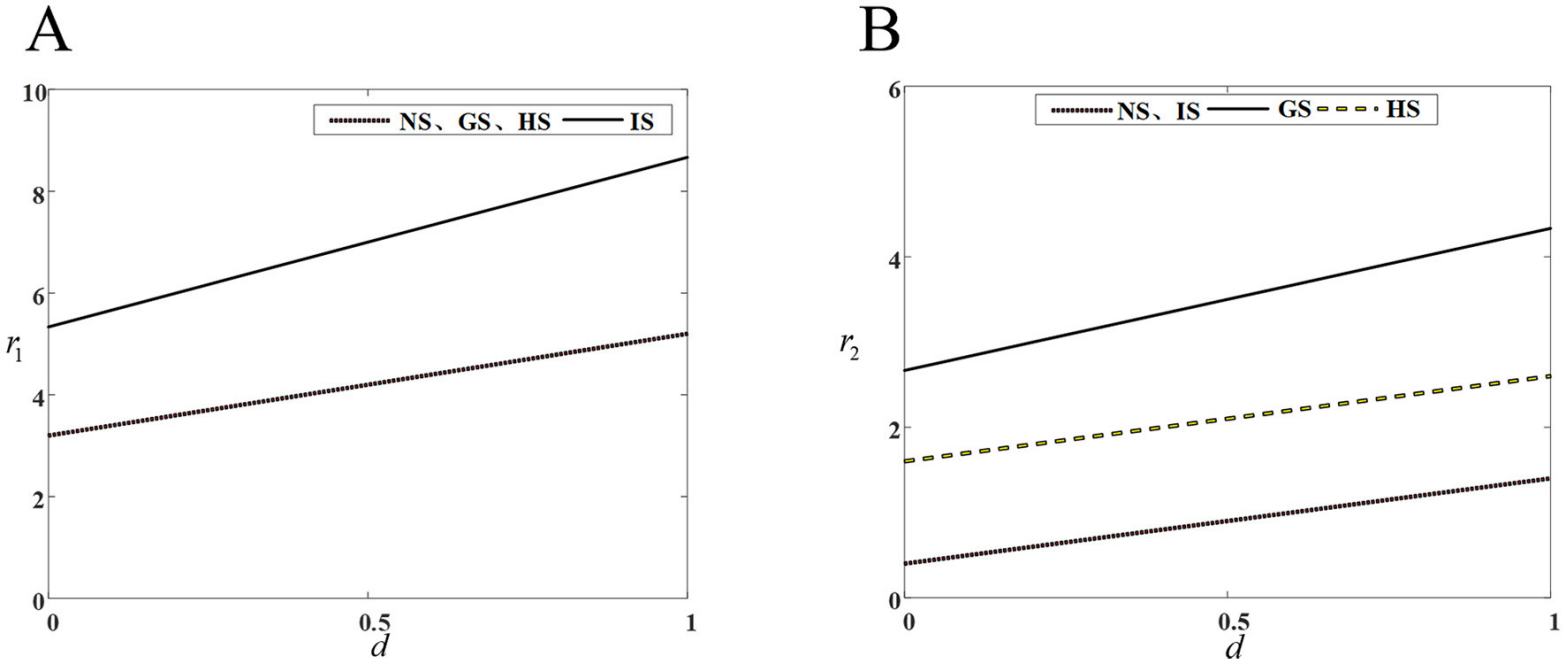
Impact of competitive intensity on drug companies' R&D investment. **(A)** R&D investment by innovative drug company; **(B)** R&D investment by generic drug company.

Figure 2 shows that innovation investment in drug R&D by innovative and generic companies in the four models, increases with the intensity of competition. Under the IS model, input decisions for innovative drugs are consistently higher than those of the other three models, while under the GS model, input decisions for generic drugs are also consistently higher than those of the other three models.

An increase competitive intensity stimulates both innovative and generic drug companies to increase their R&D investments. From a long-term perspective, generic drug companies need to enhance their investment in drug R&D and innovation to bridge the drug technology gap and either maintain or expand the market share. Simultaneously, the greater the competitive intensity, the more obvious the competitive advantage gained through drug R&D and innovation. Therefore, innovative drug companies need to improve their core competitiveness, R&D and production of innovative drugs with high technological barriers to capture a larger market share.

### 6.2 Impact of competitive intensity on drugs quality

**Figure 3:**
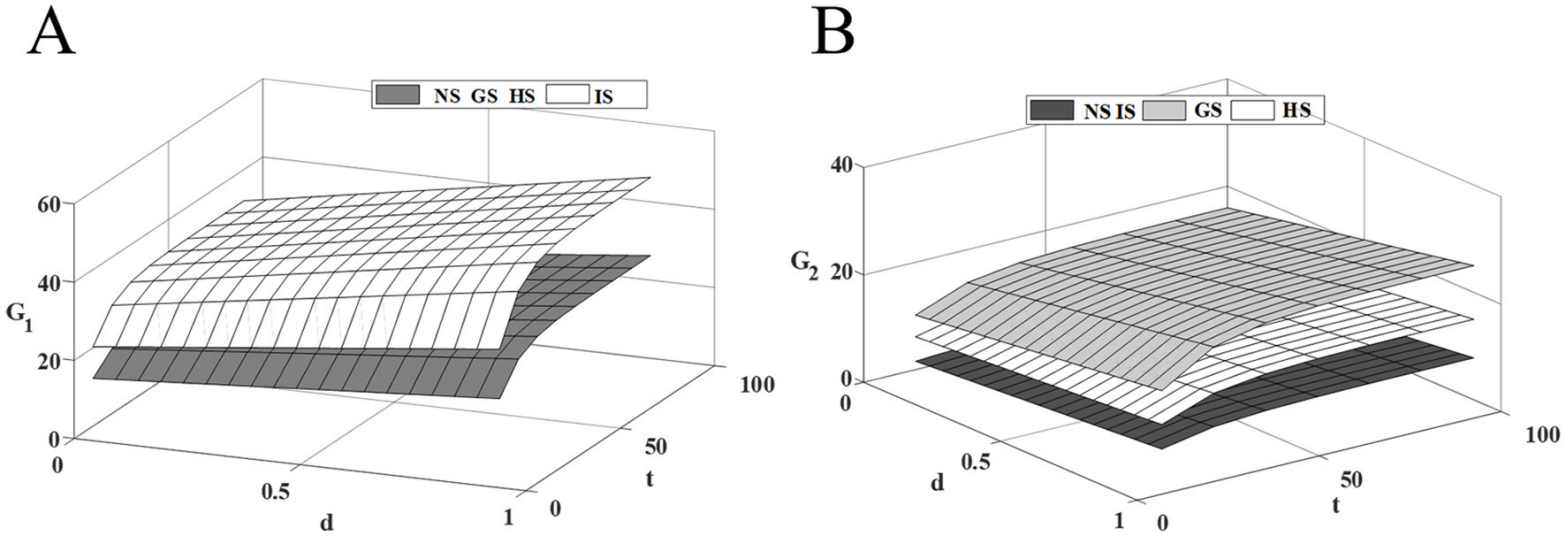
Effect of competitive intensity on drugs quality. **(A)** Innovative drug quality; **(B)** Traditional generic drug quality.

Figure 3 shows that the quality of innovative drug improves with the increase of competition intensity, and government subsidies for innovative drugs further incentivize innovative drug companies to invest in R&D costs, leading to a more rapid improvement in the quality of the drugs. However, government subsidies for generic drugs do not influence the R&D decisions of innovative drug companies, as the cost of R&D inputs for innovative drugs are more inclined to maintain their current decisions to mitigate investment risks. Therefore, innovative drug companies should improve the technical threshold of drug R&D, increase drug innovation while seeking government subsidies, expand the technological gap with similar generic drugs, and reduce the investment risk caused by the necessary update of R&D technology in the process of competition.

Both generic drug subsidies and healthcare institution subsidies contribute to the improvement of generic drug quality, but they operate on different principles. When generic drug companies receive government R&D subsidies, the cost and risk of drug R&D are effectively reduced, which encourages companies to increase R&D investment, reduce the technological gap, and capture a larger market share through higher drug quality. In contrast, healthcare institution subsidies aim to expand the market demand for generic drugs, thereby enhancing the profits of generic drug companies. And R&D investments are intended to stabilize current market share. These two incentives are distinct, with the former being more effective than the latter. Therefore, generic companies should focus on narrowing the technological gap to lessen market competition intensity, and try their best to secure government subsidies that mitigate R&D investment risks, thus ensuring the most stable drug revenue with the least cost investment.

### 6.3 Impact of competitive intensity on the profitability of supply chain members

**Figure 4:**
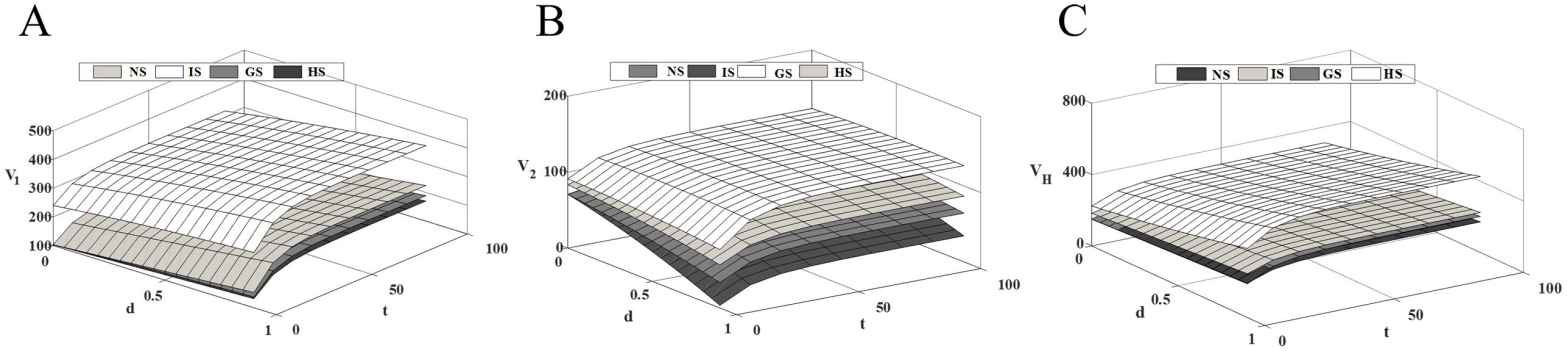
Impact of competitive intensity on supply chain members' profits. **(A)** Innovative drug company's profit; **(B)** Generic drug company's profit; **(C)** Medical institution's profit.

Figure 4 shows that as market competition intensifies, the profit level of innovative drug companies and healthcare institution continues to rise, while the profit level of generic drug companies steadily decline. As competition intensifies, the technological gap between drugs widens further. At this time, the price advantage of generic drugs in the market has been unable to make up for the technological disadvantage of the product, and patients prioritize drug efficacy over price. While government subsidies to generic drug companies can effectively alleviate this situation, they may also negatively impact the revenues of innovative drug companies. The incentive effect of the three subsidy modes on innovative and generic drug companies are opposite, while all of them have a certain degree of promotion effect on the profit enhancement of healthcare institution. Therefore, the government's subsidy strategy should consider the overall dynamics of the drug supply chain to maximize social benefits while minimizing losses.

### 6.4 Impact of competitive intensity on social welfare

**Figure 5:**
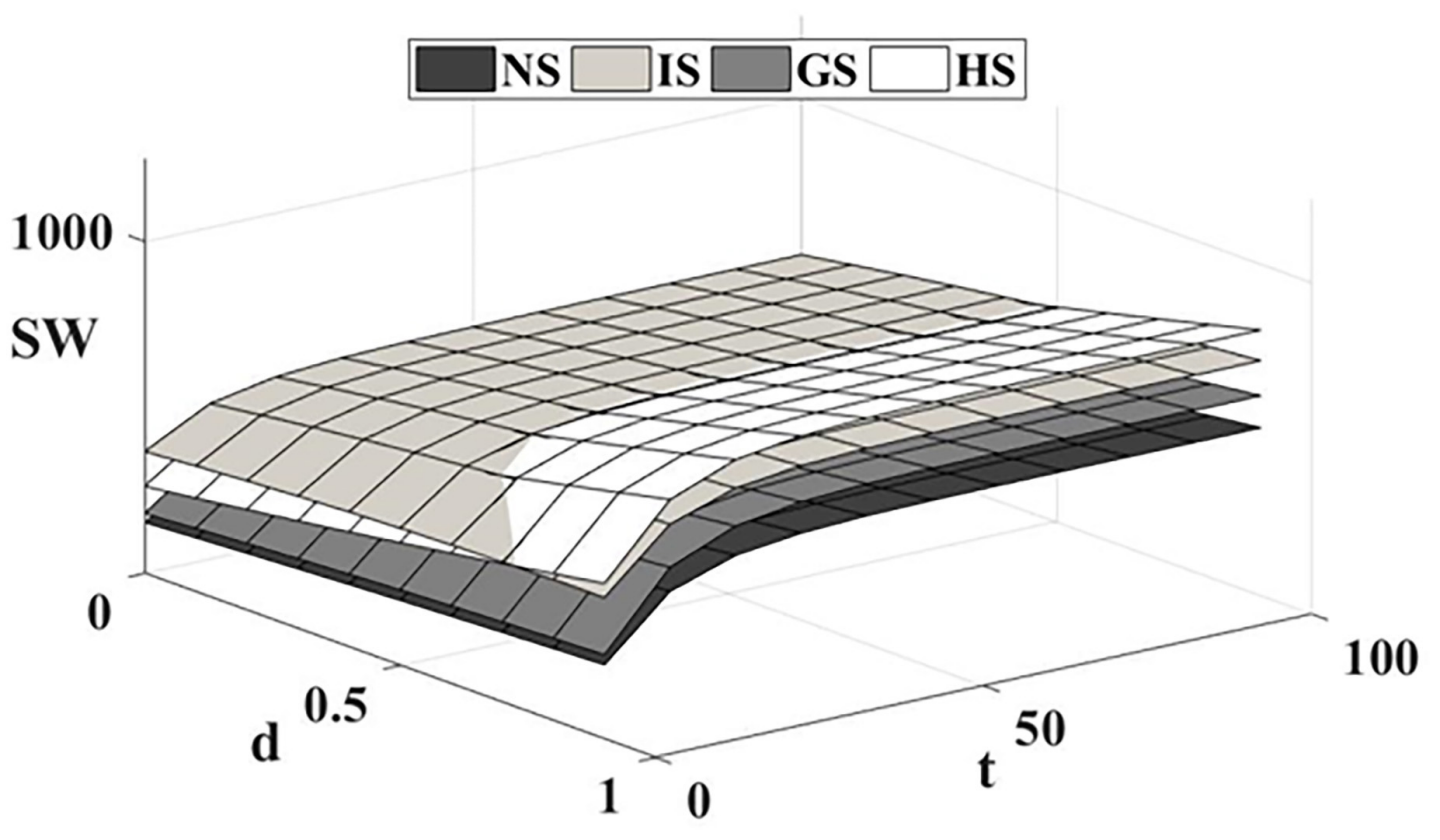
Impact of competitive intensity on social welfare.

Figure 5 shows that subsidies for drug companies and healthcare providers have a positive impact on trends in social welfare. In a low-market competitive environment, innovative drugs and generic drugs gain market demand with technological and price advantages, respectively. Government subsidies to innovative drug companies strengthen the technological advantages of innovative drugs and boost market demand. The high profitability of innovative drugs leads to a significant increase in the overall profit of the supply chain and a corresponding rise in social welfare levels. Although government subsidies to generic drug companies have also improved generic drug quality, the changes in the drug technology gap in the current environment have had a relatively small impact on demand. Consequently, the profits of both innovative and generic drug companies have not fluctuated significantly. Government subsidies to healthcare institution increased generic drug market demand, thereby increasing the profit level of generic drug companies. However, due to the price limitations of generic drugs, the overall profit level of the supply chain under this model is higher than that of the generic drug subsidy model but lower than that of the innovative drug company subsidy model.

As market competition intensifies and patients become more sensitive to technological gaps in drugs, government R&D subsidies to drug companies on one side inevitably harm the profits of companies on the other side. This effect becomes more pronounced as competition increases, causing the social welfare-enhancing effects of drug company subsidies to erode over time. And although government subsidies to healthcare institution increase the market demand for generic drugs and reduce the profits of some innovative drug companies, they are much smaller compared to the loss to the other side of the subsidy to drug companies to improve the technological gap. Moreover, the behavior of healthcare institution, in contrast, improves the overall profits of the supply chain and consumer surplus, resulting in a significantly higher level of social welfare than the drug company subsidy model. In this way, the target and proportion of government subsidies need to be reasonably adjusted according to the intensity of market competition. In low-competition intensity markets, government subsidies to innovative drug companies generate significantly higher social welfare than other subsidy models. In high-competition intensity markets, government subsidies to healthcare institution can mitigate the mutually exclusive effect of subsidies on the profits of innovative and generic drug companies, improve the overall profit level of the supply chain, and promote the reform and development of the drug industry.

### 6.5 Impact of health insurance reimbursement rates on supply chain members' profits

**Figure 6:**
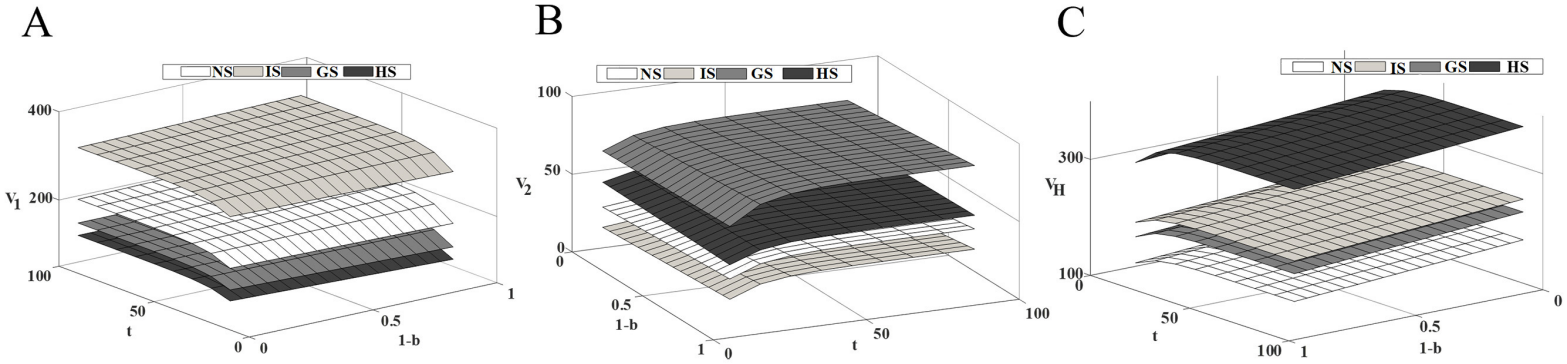
Impact of health insurance reimbursement rates on supply chain members' profits. **(A)** Innovative drug company's profit; **(B)** Generic drug company's profit; **(C)** Medical institution's profit.

Figure 6 illustrates the impact of the medical insurance reimbursement ratio on the steady-state profits of supply chain members. Under the four subsidy models, the profits of competitive drug companies and healthcare institutions increase as the reimbursement ratio rises, indicating that medical insurance policies significantly affect the economic benefits of supply chain members. As the reimbursement ratio increases, the burden on patients is reduced, the accessibility and affordability of drugs increase, thereby promoting patients' willingness to seek medical treatment and increasing the demand for drug consumption. From a long-term perspective, drug companies should increase R&D investment, improve drug quality and efficacy, capture market share, and secure greater profits. However, in addition to enhancing technological innovation and improving drug competitiveness, drug companies should also actively participate in medical insurance negotiations and include their drugs in the national medical insurance catalog. This will not only expand the market coverage of the drugs but also provide the companies with greater profit margins. Healthcare institutions should actively respond to national medical insurance policies, further implement the reimbursement scope and price adjustments for drugs, enhance the accessibility of drugs, and increase market demand.

Therefore, with the increase in the medical insurance reimbursement ratio, the interests of drug companies and healthcare institutions are intertwined, forming a positive cycle. Drug companies obtain more profits by enhancing innovation and expanding market share, while healthcare institutions improve their economic efficiency and service quality by optimizing drug procurement mechanisms and increasing operational efficiency. This interaction not only promotes the healthy development of the drug industry chain but also further enhances the overall social healthcare benefits, in line with the long-term goals of healthcare reform.

## 7 Conclusions and managerial implications

In the context of the centralized drug procurement policy, this paper constructs a two-tier competitive drug supply chain with drug companies as the central players, compares and analyzes the drug R&D strategies under four subsidy models: no government subsidy, innovative drug subsidy, generic drug subsidy, and healthcare institution subsidy, and explores the impacts of the government subsidies and market competition intensity on drug quality, supply chain decision-making, profits, and social welfare. The main conclusions drawn are as follows.

Government subsidy behavior has a significant effect on drug companies' R&D investment and healthcare institution' service cost investment. As market competition intensity increases, drug companies' enthusiasm for technological innovation and R&D investment also rises, and the government subsidies can reduce the cost and risk of companies' drug R&D. Meanwhile, government subsidies increase the income of healthcare institution and promote the reform of the income structure of public healthcare institution.

Government subsidies are more effective in enhancing the profitability and sustainability of companies at supply chain nodes. Government subsidies can significantly improve the profitability of drug companies, and providing R&D subsidies to first movers in drug development is a key part of guiding innovative drug companies toward rapid product launches, which is essential for promoting public health. In addition, government subsidies accelerate the pace of product technology upgrading and industrial transformation of generic drug companies, expanding the variety and market size of generic drugs and thus generating more profits.

The target and proportion of government subsidies should be reasonably adjusted based on the intensity of market competition. In low-competition intensity markets, government subsidies to innovative drug companies lead to significantly higher social welfare than other modes of subsidization; for high-competition intensity markets, government subsidies to healthcare institution can minimize the mutually exclusive effect of subsidies on the profits of innovative and generic drug companies, thereby promoting the reform and development of the drug industry.

In summary, drug companies and healthcare institutions should acknowledge the intense competition in the current drug market and adopt a forward-thinking approach, considering the impact of present decisions on the future. Specifically, drug companies should actively engage in research and development innovation to enhance the core competitiveness of their products and establish technological barriers in drug R&D. In drug R&D, differentiation in competition between innovative and generic drugs should be emphasized, especially under the guidance of government subsidy policies. Drug companies should increase their R&D investment, actively transform, and gradually implement a strategic shift from generic drugs to innovative drugs. Healthcare institutions should strengthen strategic cooperation with drug companies, optimize drug procurement and usage strategies, reduce drug costs, and simultaneously improve service quality. Additionally, government subsidies can assist healthcare institutions in reducing the financial burden of drugs and improving social welfare. Healthcare institutions should also adjust their cooperation models in a timely manner to respond to policy changes. For the government, subsidy policies should be designed flexibly based on market competition, supporting the development of innovative drugs while balancing the market demand for both generic and innovative drugs. Government subsidies should take into account both social welfare and the needs of various stakeholders, aiming to maximize social benefits through diversified subsidy schemes.

Our study has certain limitations and offers opportunities for future research. In our research, we treat drug production costs, drug procurement prices, and medical service prices of healthcare institutions as exogenous variables, but in reality, these factors are influenced by decision variables, which in turn affect the overall profit of the drug supply chain. Future research could consider incorporating these as control variables for more in-depth analysis. Furthermore, the study assumes that the medical service price and health insurance reimbursement ratio are the same for both innovative drugs and generic drugs. However, in reality, these rates often differ significantly between the two, and the majority of innovative drugs have yet to be included in the health insurance catalog. Therefore, future research could incorporate the endogenous factor of the impact of health insurance reimbursement, and to expand to the multi-circumstance government subsidy system.

## Data Availability

The original contributions presented in the study are included in the article/Supplementary material, further inquiries can be directed to the corresponding author.
