# Supplementary material for: Comparative study on government subsidy models for competitive drug supply chains under centralized procurement policy
Source: Front Public Health. 2025 Mar 14;13:1542858. doi: 10.3389/fpubh.2025.1542858 (PMC11949922; doi:10.3389/fpubh.2025.1542858)
Supplement: Supplementary file 1 [file Presentation_1.pdf]

frontiers

# *Appendix A*

## 1 Non-government-subsidized model (NS) proof

$$\max_{r_1} \pi_{M_1}^{\mathrm{NS}}(r_1;r_2;s_1;s_2)=\int_0^{+\infty} e^{-\rho t}\left\{w_1Q_1(t)-\frac{1}{2}r_1^2(t)\right\}\mathrm{d}t$$

$$\max_{r_2} \pi_{M_2}^{\mathrm{NS}}(r_1;r_2;s_1;s_2)=w_2Q_2(t)-\frac{1}{2}r_2^2(t)$$

$$\max_{s} \pi_{H}^{\mathrm{NS}}(r_1;r_2;s_1;s_2)=\int_0^{+\infty} e^{-\rho t}\left\{p\left[Q_1(t)+Q_2(t)\right]-\frac{1}{2}s_1^2(t)-\frac{1}{2}s_2^2(t)\right\}\mathrm{d}t$$

$$\text{s.t.}\quad\begin{cases}\dot{G}_i(t)=\mu r_i(t)-kG_i(t)\\ \dot{G}_i(0)=G_0,\quad i=1,2\end{cases}$$

$$\rho V_{\mathrm{M}_1}^{\mathrm{NS}}=\left\{w_1\left[\varepsilon a-bw_1+\alpha r_1+\beta s_1+\lambda G_1+d\left(r_1-r_2\right)\right]-\frac{1}{2}r_1^2+\frac{\partial V_{\mathrm{M}_1}^{\mathrm{NS}}}{\partial G_1}\left(\mu r_1-kG_1\right)\right\}$$

$$\rho V_{\mathrm{M}_2}^{\mathrm{NS}}=\left\{w_2\left[\left(1-\varepsilon\right)a-bw_2+\alpha r_2+\beta s_2+\lambda G_2+d\left(r_2-r_1\right)\right]-\frac{1}{2}r_2^2\right\}$$

$$\rho V_{\mathrm{H}}^{\mathrm{NS}}=\left\{\begin{array}{l}p\left[a-b\left(w_1+w_2\right)+\alpha\left(r_1+r_2\right)+\beta\left(s_1+s_2\right)+\lambda\left(G_1+G_2\right)\right]\\ -\frac{1}{2}s_1^2-\frac{1}{2}s_2^2+\frac{\partial V_H^{\mathrm{NS}}}{\partial G_1}\left(\mu r_1-kG_1\right)+\frac{\partial V_H^{\mathrm{NS}}}{\partial G_2}\left(\mu r_2-kG_2\right)\end{array}\right\}$$

The first-order optimality conditions yield:

$$r_1=w_1\left(\alpha+d\right)+\frac{\partial V_{\mathrm{M}_1}^{\mathrm{NS}}}{\partial G_1}\mu,\quad r_2=w_2\left(\alpha+d\right) \qquad \text{(A-3)}$$

$$s_1=p\beta,\quad s_2=p\beta$$

Bringing (A-3) into (A-2) yields

$$\rho V_{\mathrm{M}_1}^{\mathrm{NS}}=\begin{Bmatrix}\left(w_1\lambda-k\frac{\partial V_{\mathrm{M}_1}^{\mathrm{NS}}}{\partial G_1}\right)G_1-\frac{1}{2}\left[w_1\left(\alpha+d\right)+\mu\frac{\partial V_{\mathrm{M}_1}^{\mathrm{NS}}}{\partial G_1}\right]^2+\frac{\partial V_{\mathrm{M}_1}^{\mathrm{NS}}}{\partial G_1}\left(\mu w_1\left(\alpha+d\right)+\mu^2\frac{\partial V_{\mathrm{M}_1}^{\mathrm{NS}}}{\partial G_1}\right)\\+w_1\left[\varepsilon a-bw_1+\alpha w_1\left(\alpha+d\right)+\alpha\mu\frac{\partial V_{\mathrm{M}_1}^{\mathrm{NS}}}{\partial G_1}+p\beta^2+d\left(w_1-w_2\right)\left(\alpha+d\right)+d\mu\frac{\partial V_{\mathrm{M}_1}^{\mathrm{NS}}}{\partial G_1}\right]\end{Bmatrix}$$

$$\rho V_{\mathrm{M}_2}^{\mathrm{NS}}=\left\{w_2\lambda G_2+w_2\left[\left(1-\varepsilon\right)a-bw_2+\alpha w_2\left(\alpha+d\right)+p\beta^2+d\left(w_2-w_1\right)\left(\alpha+d\right)-d\mu\frac{\partial V_{\mathrm{M}_1}^{\mathrm{NS}}}{\partial G_1}\right]-\frac{1}{2}w_2^2\left(\alpha+d\right)^2\right\}$$

$$\rho V_{\mathrm{H}}^{\mathrm{NS}}=\begin{Bmatrix}\left(p\lambda-k\frac{\partial V_{\mathrm{H}}^{\mathrm{NS}}}{\partial G_1}\right)G_1+\left(p\lambda-k\frac{\partial V_{\mathrm{H}}^{\mathrm{NS}}}{\partial G_2}\right)G_2\\+p\left[a-b\left(\theta w_1+w_2\right)+\alpha\left(\theta w_1+w_2\right)\left(\alpha+d\right)+\alpha\mu\frac{\partial V_{\mathrm{M}_1}^{\mathrm{NS}}}{\partial G_1}\right]\\+p^2\beta^2+\frac{\partial V_{\mathrm{H}}^{\mathrm{NS}}}{\partial G_1}\left[\mu\theta w_1\left(\alpha+d\right)+\mu^2\frac{\partial V_{\mathrm{M}_1}^{\mathrm{NS}}}{\partial G_1}\right]+\mu w_2\left(\alpha+d\right)\frac{\partial V_{\mathrm{H}}^{\mathrm{NS}}}{\partial G_2}\end{Bmatrix}$$

Based on the structure of the above equation, the optimal value function of the three parties is assumed to be:

$V_{\mathrm{M}_1}^{\mathrm{NS}}=m_1G_1+l_1, V_{\mathrm{M}_2}^{\mathrm{NS}}=n_1G_2+l_2, V_{\mathrm{H}}^{\mathrm{NS}}=x_1G_1+y_1G_2+l_3$

Use the constant relationship to identify unrecognized parameters as:

$$m_1=\frac{w_1\lambda}{\rho+k}$$

$$l_1=\frac{w_1}{\rho}\left[\varepsilon a-bw_1+p\beta^2-\left(\alpha+d\right)dw_2\right]+\frac{1}{2\rho}\left[w_1\left(\alpha+d\right)+\mu\frac{w_1\lambda}{\rho+k}\right]^2$$

$$n_1=\frac{w_2\lambda}{\rho}$$

$$l_2=\frac{w_2}{\rho}\left[\left(1-\varepsilon\right)a-bw_2+p\beta^2-\left(\alpha+d\right)dw_1-\frac{d\mu w_1\lambda}{\rho+k}\right]+\frac{1}{2\rho}w_2^2\left(\alpha+d\right)^2$$

$$x_1=\frac{p\lambda}{\rho+k}$$

$$y_1=\frac{p\lambda}{\rho+k}$$

$$l_3=\frac{1}{\rho}\begin{Bmatrix}p\left[a-\left(w_1+w_2\right)b+\left(w_1+w_2\right)\alpha\left(\alpha+d\right)\right]+p^2\beta^2\\+\mu\frac{w_1\lambda}{\rho+k}\left(p\alpha+\mu\frac{p\lambda}{\rho+k}\right)+\left(\alpha+d\right)\left(w_1+w_2\right)\mu\frac{p\lambda}{\rho+k}\end{Bmatrix}$$

End of proof.

## 2 Innovative drug subsidy model (IS) proof

$$\max_{r_1} \pi_{\mathrm{M}_1}^{\mathrm{IS}}(r_1;r_2;s_1;s_2)=\int_0^{+\infty} e^{-\rho t}\left\{w_1 Q_1(t)-\frac{1}{2}(1-\theta)r_1^2(t)\right\}\mathrm{d}t$$

$$\max_{r_2} \pi_{\mathrm{M}_2}^{\mathrm{IS}}(r_1;r_2;s_1;s_2)=w_2 Q_2(t)-\frac{1}{2}r_2^2(t)$$

$$\max_{s} \pi_{\mathrm{H}}^{\mathrm{IS}}(r_1;r_2;s_1;s_2)=\int_0^{+\infty} e^{-\rho t}\left\{p\left[Q_1(t)+Q_2(t)\right]-\frac{1}{2}s_1^2(t)-\frac{1}{2}s_2^2(t)\right\}\mathrm{d}t \tag{A-4}$$

$$\text{s.t. } \begin{cases}\dot{G}_i(t)=\mu r_i(t)-kG_i(t)\\ \dot{G}_i(0)=G_0,\quad i=1,2\end{cases}$$

$$\rho V_{\mathrm{M}_1}^{\mathrm{IS}}=\left\{w_1\left[\varepsilon a-bw_1+\alpha r_1+\beta s_1+\lambda G_1+d\left(r_1-r_2\right)\right]-\frac{1}{2}(1-\theta)r_1^2+\frac{\partial V_{\mathrm{M}_1}^{\mathrm{IS}}}{\partial G_1}\left(\mu r_1-kG_1\right)\right\}$$

$$\rho V_{\mathrm{M}_2}^{\mathrm{IS}}=\left\{w_2\left[\left(1-\varepsilon\right)a-bw_2+\alpha r_2+\beta s_2+\lambda G_2+d\left(r_2-r_1\right)\right]-\frac{1}{2}r_2^2\right\} \tag{A-5}$$

$$\rho V_{\mathrm{H}}^{\mathrm{IS}}=\begin{Bmatrix}p\left[a-b\left(w_1+w_2\right)+\alpha\left(r_1+r_2\right)+\beta\left(s_1+s_2\right)+\lambda\left(G_1+G_2\right)\right]\\ -\frac{1}{2}s_1^2-\frac{1}{2}s_2^2+\frac{\partial V_H^{\mathrm{IS}}}{\partial G_1}\left(\mu r_1-kG_1\right)+\frac{\partial V_H^{\mathrm{IS}}}{\partial G_2}\left(\mu r_2-kG_2\right)\end{Bmatrix}$$

The first-order optimality conditions yield:

$$r_1=\frac{1}{(1-\theta)}\left[w_1\left(\alpha+d\right)+\mu\frac{\partial V_{\mathrm{M}_1}^{\mathrm{IS}}}{\partial G_1}\right],\quad r_2=w_2\left(\alpha+d\right)$$

$$s_1=p\beta,\quad s_2=p\beta \tag{A-6}$$

(A-6) carried over into (A-5) yields

$$\rho V_{\mathrm{M}_1}^{\mathrm{IS}}=\begin{Bmatrix}\left(w_1\lambda-k\frac{\partial V_{\mathrm{M}_1}^{\mathrm{IS}}}{\partial G_1}\right)G_1+w_1\left[\varepsilon a-bw_1+p\beta^2+\frac{(\alpha+d)}{(1-\theta)}\left[w_1\left(\alpha+d\right)+\mu\frac{\partial V_{\mathrm{M}_1}^{\mathrm{IS}}}{\partial G_1}\right]-dw_2\left(\alpha+d\right)\right]\\ -\frac{1}{2(1-\theta)}\left[w_1\left(\alpha+d\right)+\mu\frac{\partial V_{\mathrm{M}_1}^{\mathrm{IS}}}{\partial G_1}\right]^2+\frac{\mu}{(1-\theta)}\left[w_1\left(\alpha+d\right)+\mu\frac{\partial V_{\mathrm{M}_1}^{\mathrm{IS}}}{\partial G_1}\right]\frac{\partial V_{\mathrm{M}_1}^{\mathrm{IS}}}{\partial G_1}\end{Bmatrix}$$

$$\rho V_{\mathrm{M}_2}^{\mathrm{IS}}=\begin{Bmatrix}w_2\lambda G_2-\frac{1}{2}w_2^2\left(\alpha+d\right)^2\\ +w_2\left[\left(1-\varepsilon\right)a-bw_2+p\beta^2+w_2\left(\alpha+d\right)^2-\frac{w_1 d}{(1-\theta)}\left(\alpha+d\right)-\frac{\mu d}{(1-\theta)}\frac{\partial V_{\mathrm{M}_1}^{\mathrm{IS}}}{\partial G_1}\right]\end{Bmatrix}$$

$$\rho V_{\mathrm{H}}^{\mathrm{IS}}=\begin{Bmatrix}\left(p\lambda-k\frac{\partial V_H^{\mathrm{IS}}}{\partial G_1}\right)G_1-\left(p\lambda-k\frac{\partial V_H^{\mathrm{IS}}}{\partial G_2}\right)G_2\\ p\left[a-b\left(w_1+w_2\right)+\frac{\alpha w_1}{(1-\theta)}\left(\alpha+d\right)+\alpha w_2\left(\alpha+d\right)+\frac{\alpha\mu}{(1-\theta)}\frac{\partial V_{\mathrm{M}_1}^{\mathrm{IS}}}{\partial G_1}\right]\\ +p^2\beta^2+\mu\frac{1}{(1-\theta)}\left[w_1\left(\alpha+d\right)+\mu\frac{\partial V_{\mathrm{M}_1}^{\mathrm{IS}}}{\partial G_1}\right]\frac{\partial V_H^{\mathrm{GS}}}{\partial G_1}+\mu w_2\left(\alpha+d\right)\frac{\partial V_H^{\mathrm{IS}}}{\partial G_2}\end{Bmatrix}$$

Based on the structure of the above equation, the optimal value function of the three parties is assumed to be:

$V_{M_1}^{IS} = m_2 G_1 + l_4, V_{M_2}^{IS} = n_2 G_2 + l_5, V_H^{IS} = x_2 G_1 + y_2 G_2 + l_6$

Use the identity relation to determine the unacknowledged parameters as:

$$m_2 = \frac{w_1 \lambda}{k+\rho}$$

$$l_4 = \frac{w_1}{\rho}\left[\varepsilon a - b w_1 + p\beta^2 - d w_2(\alpha+d)\right] + \frac{1}{2\rho(1-\theta)}\left[w_1(\alpha+d) + \mu\frac{w_1\lambda}{k+\rho}\right]^2$$

$$n_2 = \frac{w_2 \lambda}{\rho}$$

$$l_5 = \frac{w_2}{\rho}\left[(1-\varepsilon)a - b w_2 + p\beta^2 - \frac{w_1 d}{(1-\theta)}(\alpha+d) - \frac{\mu d w_1 \lambda}{(1-\theta)(\rho+k)}\right] + \frac{1}{2\rho} w_2{}^2 (\alpha+d)^2$$

$$x_2 = \frac{p\lambda}{\rho+k}$$

$$y_2 = \frac{p\lambda}{\rho+k}$$

$$l_6 = \frac{1}{\rho}\left\{\begin{array}{l} p\left[a - b(w_1+w_2) + \alpha(\alpha+d)\left(\frac{w_1}{1-\theta}+w_2\right)\right] + p^2\beta^2 \\ +\frac{\mu w_1 \lambda}{(\rho+k)(1-\theta)}\left(p\alpha + \mu\frac{p\lambda}{\rho+k}\right) + (\alpha+d)\frac{\mu p \lambda}{\rho+k}\left(\frac{w_1}{1-\theta}+w_2\right)\end{array}\right\}$$

End of proof.

## 3 Generic drug subsidy model (GS) proof

$$\max_{r_1} \pi_{M_1}^{GS}(r_1; r_2; s_1; s_2) = \int_0^{+\infty} e^{-\rho t}\left\{w_1 Q_1(t) - \frac{1}{2} r_1^2(t)\right\}dt$$

$$\max_{r_2} \pi_{M_2}^{GS}(r_1; r_2; s_1; s_2) = \int_0^{+\infty} e^{-\rho t}\left\{w_2 Q_2(t) - \frac{1}{2}(1-\delta) r_2^2(t)\right\}dt$$

$$\max_{s} \pi_{H}^{GS}(r_1; r_2; s_1; s_2) = \int_0^{+\infty} e^{-\rho t}\left\{p\left[Q_1(t) + Q_2(t)\right] - \frac{1}{2}s_1^2(t) - \frac{1}{2}s_2^2(t)\right\}dt \quad \text{(A-7)}$$

$$\text{s.t.} \quad \begin{cases} \dot{G}_1(t) = \mu r_1(t) - k G_1(t), G_1(0) = G_0 \\ \dot{G}_2(t) = \mu r_2(t) - k G_2(t), G_2(0) = G_0 \end{cases}$$

$$\rho V_{M_1}^{GS}=\left\{w_1\left[\varepsilon a-bw_1+\alpha r_1+\beta s_1+\lambda G_1+d\left(r_1-r_2\right)\right]-\frac{1}{2}r_1^2+\frac{\partial V_{M_1}^{GS}}{\partial G_1}\left(\mu r_1-kG_1\right)\right\}$$

$$\rho V_{M_2}^{GS}=\left\{\begin{array}{l} w_2\left[\left(1-\varepsilon\right)a-bw_2+\alpha r_2+\beta s_2+\lambda G_2+d\left(r_2-r_1\right)\right] \\ -\frac{1}{2}\left(1-\delta\right)r_2^2+\frac{\partial V_{M_2}^{GS}}{\partial G_2}\left(\mu r_2-kG_2\right) \end{array}\right\} \tag{A-8}$$

$$\rho V_{H}^{GS}=\left\{\begin{array}{l} p\left[a-b\left(w_1+w_2\right)+\alpha\left(r_1+r_2\right)+\beta\left(s_1+s_2\right)+\lambda\left(G_1+G_2\right)\right] \\ -\frac{1}{2}s_1{}^2-\frac{1}{2}s_2{}^2+\frac{\partial V_{H}^{GS}}{\partial G_1}\left(\mu r_1-kG_1\right)+\frac{\partial V_{H}^{GS}}{\partial G_2}\left(\mu r_2-kG_2\right) \end{array}\right\}$$

The first-order optimality conditions yield:

$$r_1=w_1\left(\alpha+d\right)+\mu\frac{\partial V_{M_1}^{GS}}{\partial G_1},\quad r_2=\frac{1}{\left(1-\delta\right)}\left[w_2\left(\alpha+d\right)+\mu\frac{\partial V_{M_2}^{GS}}{\partial G_2}\right] \tag{A-9}$$

$$s_1=p\beta,\quad s_2=p\beta$$

(A-9) carried over into (A-8) gives:

$$\rho V_{M_1}^{GS}=\left\{\begin{array}{l} \left(w_1\lambda-k\frac{\partial V_{M_1}^{GS}}{\partial G_1}\right)G_1-\frac{1}{2}\left[w_1\left(\alpha+d\right)+\mu\frac{\partial V_{M_1}^{GS}}{\partial G_1}\right]^2+\frac{\partial V_{M_1}^{GS}}{\partial G_1}\left[\mu w_1\left(\alpha+d\right)+\mu^2\frac{\partial V_{M_1}^{GS}}{\partial G_1}\right] \\ +w_1\left[\begin{array}{l} \varepsilon a-bw_1+\alpha w_1\left(\alpha+d\right)+\alpha\mu\frac{\partial V_{M_1}^{GS}}{\partial G_1}+p\beta^2 \\ +d\left(\alpha+d\right)\left(w_1-\frac{w_2}{1-\delta}\right)+\mu d\left(\frac{\partial V_{M_1}^{GS}}{\partial G_1}-\frac{1}{1-\delta}\frac{\partial V_{M_2}^{GS}}{\partial G_2}\right) \end{array}\right] \end{array}\right\}$$

$$\rho V_{M_2}^{GS}=\left\{\begin{array}{l} \left(w_2\lambda-k\frac{\partial V_{M_2}^{GS}}{\partial G_2}\right)G_2-\frac{1}{2\left(1-\delta\right)}\left[w_2\left(\alpha+d\right)+\mu\frac{\partial V_{M_2}^{GS}}{\partial G_2}\right]^2+\frac{\partial V_{M_2}^{GS}}{\partial G_2}\frac{\mu}{\left(1-\delta\right)}\left[w_2\left(\alpha+d\right)+\mu\frac{\partial V_{M_2}^{GS}}{\partial G_2}\right] \\ w_2\left[\begin{array}{l} \left(1-\varepsilon\right)a-bw_2+\frac{\alpha w_2}{\left(1-\delta\right)}\left(\alpha+d\right)+\frac{\alpha\mu}{\left(1-\delta\right)}\frac{\partial V_{M_2}^{GS}}{\partial G_2} \\ +p\beta^2+d\left(\alpha+d\right)\left(\frac{w_2}{1-\delta}-w_1\right)+\mu d\left(\frac{1}{1-\delta}\frac{\partial V_{M_2}^{GS}}{\partial G_2}-\frac{\partial V_{M_1}^{GS}}{\partial G_1}\right) \end{array}\right] \end{array}\right\}$$

$$\rho V_{H}^{GS}=\left\{\begin{array}{l} \left(\lambda p-k\frac{\partial V_{H}^{GS}}{\partial G_1}\right)G_1+\left(\lambda p-k\frac{\partial V_{H}^{GS}}{\partial G_2}\right)G_2+p^2\beta^2 \\ p\left[a-b\left(w_1+w_2\right)+\alpha\left(\alpha+d\right)\left(w_1+\frac{w_2}{1-\delta}\right)+\alpha\mu\left(\frac{\partial V_{M_1}^{GS}}{\partial G_1}+\frac{1}{\left(1-\delta\right)}\frac{\partial V_{M_2}^{GS}}{\partial G_2}\right)\right] \\ +\mu\frac{\partial V_{H}^{GS}}{\partial G_1}\left(w_1\left(\alpha+d\right)+\mu^2\frac{\partial V_{M_1}^{GS}}{\partial G_1}\right)+\frac{\mu}{\left(1-\delta\right)}\frac{\partial V_{H}^{GS}}{\partial G_2}\left[w_2\left(\alpha+d\right)+\mu\frac{\partial V_{M_2}^{GS}}{\partial G_2}\right] \end{array}\right\}$$

Based on the structure of the above equation, the optimal value function of the three parties is assumed to be:

$$V_{M_1}^{GS}=m_3G_1+l_7,V_{M_2}^{GS}=n_3G_2+l_8,V_{H}^{GS}=x_3G_1+y_3G_2+l_9$$

Use the identity relation to determine the unacknowledged parameters as:

$$m_3 = \frac{w_1\lambda}{\rho+k}$$

$$l_7 = \frac{w_1}{\rho}\left[\varepsilon a - bw_1 + p\beta^2 - d\left(\alpha+d\right)\frac{w_2}{1-\delta} - \frac{\mu d w_2\lambda}{\left(\rho+k\right)\left(1-\delta\right)}\right] + \frac{1}{2\rho}\left[w_1\left(\alpha+d\right)+\mu\frac{w_1\lambda}{\rho+k}\right]^2$$

$$n_3 = \frac{w_2\lambda}{\rho+k}$$

$$l_8 = \frac{w_2}{\rho}\left[\left(1-\varepsilon\right)a - bw_2 + p\beta^2 - d\left(\alpha+d\right)w_1 - \frac{\mu d w_1\lambda}{\rho+k}\right] + \frac{1}{2\left(1-\delta\right)\rho}\left[w_2\left(\alpha+d\right)+\mu\frac{w_2\lambda}{\rho+k}\right]^2$$

$$x_3 = \frac{\lambda p}{\rho+k}$$

$$y_3 = \frac{\lambda p}{\rho+k}$$

$$l_9 = \frac{1}{\rho}\left\{\begin{array}{l} p\left[a-b\left(w_1+w_2\right)+\alpha\left(w_1+\frac{w_2}{1-\delta}\right)\left(\alpha+d+\frac{\mu\lambda}{\rho+k}\right)\right]+p^2\beta^2 \\ +\frac{\mu\lambda p}{\rho+k}\left[\left(w_1+\frac{w_2}{1-\delta}\right)\left(\alpha+d\right)+\frac{\mu\lambda}{\rho+k}\left(\mu w_1+\frac{w_2}{1-\delta}\right)\right] \end{array}\right\}$$

End of proof.

## 4 Healthcare institution subsidy model (HS) proof

$$\begin{aligned}
&\max_{r_1}\pi_{\mathrm{M}_1}^{\mathrm{HS}}(r_1;r_2;s_1;s_2) = \int_0^{+\infty} e^{-\rho t}\left\{w_1 Q_1\left(t\right) - \frac{1}{2}r_1^2\left(t\right)\right\}\mathrm{d}t \\
&\max_{r_2}\pi_{\mathrm{M}_2}^{\mathrm{HS}}(r_1;r_2;s_1;s_2) = \int_0^{+\infty} e^{-\rho t}\left\{w_2 Q_2\left(t\right) - \frac{1}{2}r_2^2\left(t\right)\right\}\mathrm{d}t \\
&\max_{s}\pi_{\mathrm{H}}^{\mathrm{HS}}(r_1;r_2;s_1;s_2) = \int_0^{+\infty} e^{-\rho t}\left\{pQ_1\left(t\right) + (p+\eta w_2)Q_2\left(t\right) - \frac{1}{2}s_1^2\left(t\right) - \frac{1}{2}s_2^2\left(t\right)\right\}\mathrm{d}t \qquad \text{(A-10)} \\
&\text{s.t.}\ \begin{cases} \dot{G}_1(t) = \mu r_1(t) - kG_1(t), G_1\left(0\right) = G_0 \\ \dot{G}_2(t) = \mu r_2(t) - kG_2(t), G_2\left(0\right) = G_0 \end{cases}
\end{aligned}$$

$$\rho V_{\mathrm{M}_1}^{\mathrm{HS}} = \left\{ w_1\left[\varepsilon a - bw_1 + \alpha r_1 + \beta s_1 + \lambda G_1 + d\left(r_1 - r_2\right)\right] - \frac{1}{2}r_1^2 + \frac{\partial V_{\mathrm{M}_1}^{\mathrm{HS}}}{\partial G_1}\left(\mu r_1 - kG_1\right) \right\}$$

$$\rho V_{\mathrm{M}_2}^{\mathrm{HS}} = \left\{ w_2\left[\left(1-\varepsilon\right)a - bw_2 + \alpha r_2 + \beta s_2 + \lambda G_2 + d\left(r_2 - r_1\right)\right] - \frac{1}{2}r_2^2 + \frac{\partial V_{\mathrm{M}_2}^{\mathrm{HS}}}{\partial G_2}\left(\mu r_2 - kG_2\right) \right\} \quad \text{(A-11)}$$

$$\rho V_{\mathrm{H}}^{\mathrm{HS}} = \left\{ \begin{array}{l} p\left[a - b\left(w_1 + w_2\right) + \alpha\left(r_1 + r_2\right) + \beta\left(s_1 + s_2\right) + \lambda\left(G_1 + G_2\right)\right] \\ +\eta w_2\left[\left(1-\varepsilon\right)a - bw_2 + \alpha r_2 + \beta s_2 + \lambda G_2 + d\left(r_2 - r_1\right)\right] \\ -\frac{1}{2}s_1{}^2 - \frac{1}{2}s_2{}^2 + \frac{\partial V_H^{\mathrm{HS}}}{\partial G_1}\left(\mu r_1 - kG_1\right) + \frac{\partial V_H^{\mathrm{HS}}}{\partial G_2}\left(\mu r_2 - kG_2\right) \end{array} \right\}$$

The first-order optimality conditions yield:

$$r_1 = w_1\left(\alpha + d\right) + \mu\frac{\partial V_{\mathrm{M}_1}^{\mathrm{HS}}}{\partial G_1}, \quad r_2 = w_2\left(\alpha + d\right) + \mu\frac{\partial V_{\mathrm{M}_2}^{\mathrm{HS}}}{\partial G_2}$$

$$s_1 = p\beta, \quad s_2 = \left(p + \eta w_2\right)\beta \quad \text{(A-12)}$$

(A-12) is brought into (A-11) to obtain:

$$\rho V_{\mathrm{M}_1}^{\mathrm{HS}} = \left\{ \begin{array}{l} \left(w_1\lambda - k\frac{\partial V_{\mathrm{M}_1}^{\mathrm{HS}}}{\partial G_1}\right)G_1 - \frac{1}{2}\left[w_1\left(\alpha + d\right) + \mu\frac{\partial V_{\mathrm{M}_1}^{\mathrm{HS}}}{\partial G_1}\right]^2 + \frac{\partial V_{\mathrm{M}_1}^{\mathrm{HS}}}{\partial G_1}\left(\mu w_1\left(\alpha + d\right) + \mu^2\frac{\partial V_{\mathrm{M}_1}^{\mathrm{HS}}}{\partial G_1}\right) \\ +w_1\left[\varepsilon a - bw_1 + \alpha w_1\left(\alpha + d\right) + \alpha\mu\frac{\partial V_{\mathrm{M}_1}^{\mathrm{HS}}}{\partial G_1} + p\beta^2 + d\left(\alpha + d\right)\left(w_1 - w_2\right) + \mu d\left(\frac{\partial V_{\mathrm{M}_1}^{\mathrm{HS}}}{\partial G_1} - \frac{\partial V_{\mathrm{M}_2}^{\mathrm{HS}}}{\partial G_2}\right)\right] \end{array} \right\}$$

$$\rho V_{\mathrm{M}_2}^{\mathrm{HS}} = \left\{ \begin{array}{l} \left(w_2\lambda - k\frac{\partial V_{\mathrm{M}_2}^{\mathrm{HS}}}{\partial G_2}\right)G_2 - \frac{1}{2}w_2\left[\left(\alpha + d\right) + \mu\frac{\partial V_{\mathrm{M}_2}^{\mathrm{HS}}}{\partial G_2}\right]^2 + \frac{\partial V_{\mathrm{M}_2}^{\mathrm{HS}}}{\partial G_2}\left(\mu w_2\left(\alpha + d\right) + \mu^2\frac{\partial V_{\mathrm{M}_2}^{\mathrm{HS}}}{\partial G_2}\right) \\ w_2\left[\left(1-\varepsilon\right)a - bw_2 + \alpha w_2\left(\alpha + d\right) + \alpha\mu\frac{\partial V_{\mathrm{M}_2}^{\mathrm{HS}}}{\partial G_2} + \left(p + \eta w_2\right)\beta^2 + d\left(\alpha + d\right)\left(w_2 - w_1\right) + \mu d\left(\frac{\partial V_{\mathrm{M}_2}^{\mathrm{HS}}}{\partial G_2} - \frac{\partial V_{\mathrm{M}_1}^{\mathrm{HS}}}{\partial G_1}\right)\right] \end{array} \right\}$$

$$\rho V_{\mathrm{H}}^{\mathrm{HS}} = \left\{ \begin{array}{l} \left(\lambda p - k\frac{\partial V_H^{\mathrm{HS}}}{\partial G_1}\right)G_1 + \left[\lambda\left(p + \eta w_2\right) - k\frac{\partial V_H^{\mathrm{HS}}}{\partial G_2}\right]G_2 \\ +p\left[a - b\left(w_1 + w_2\right) + \alpha\left(\alpha + d\right)\left(w_1 + w_2\right) + \alpha\mu\left(\frac{\partial V_{\mathrm{M}_1}^{\mathrm{HS}}}{\partial G_1} + \frac{\partial V_{\mathrm{M}_2}^{\mathrm{HS}}}{\partial G_2}\right)\right] \\ +\eta w_2\left[\left(1-\varepsilon\right)a - bw_2 + \alpha w_2\left(\alpha + d\right) + \mu\frac{\partial V_{\mathrm{M}_2}^{\mathrm{HS}}}{\partial G_2} + \left(p + \eta w_2\right)\beta^2 + d\left(\alpha + d\right)\left(w_2 - w_1\right) + \mu d\left(\frac{\partial V_{\mathrm{M}_2}^{\mathrm{HS}}}{\partial G_2} - \frac{\partial V_{\mathrm{M}_1}^{\mathrm{HS}}}{\partial G_1}\right)\right] \\ +\frac{3}{2}p^2\beta^2 - \frac{1}{2}\left(p + \eta w_2\right)^2\beta^2 + \eta w_2 p\beta^2 + \mu\left(\alpha + d\right)\left(w_1\frac{\partial V_H^{\mathrm{HS}}}{\partial G_1} + w_2\frac{\partial V_H^{\mathrm{HS}}}{\partial G_2}\right) + \mu^2\frac{\partial V_H^{\mathrm{HS}}}{\partial G_1}\frac{\partial V_{\mathrm{M}_1}^{\mathrm{HS}}}{\partial G_1} + \mu^2\frac{\partial V_H^{\mathrm{HS}}}{\partial G_2}\frac{\partial V_{\mathrm{M}_2}^{\mathrm{HS}}}{\partial G_2} \end{array} \right\}$$

Based on the structure of the above equation, the optimal value function of the three parties is assumed to be:

$V_{\mathrm{M}_1}^{\mathrm{HS}} = m_4G_1 + l_{10}, V_{\mathrm{M}_2}^{\mathrm{GS}} = n_4G_2 + l_{11}, V_{\mathrm{H}}^{\mathrm{GS}} = x_4G_1 + y_4G_2 + l_{12}$

Use the identity relation to determine the unacknowledged parameters as:

$$m_4 = \frac{w_1 \lambda}{\rho + k}$$

$$l_{10} = \frac{w_1}{\rho}\left[\varepsilon a - bw_1 + p\beta^2 - d\left(\alpha + d\right)w_2 - \frac{\mu d w_2 \lambda}{\rho + k}\right] + \frac{1}{2\rho}\left[w_1\left(\alpha + d\right) + \mu\frac{w_1 \lambda}{\rho + k}\right]^2$$

$$n_4 = \frac{w_2 \lambda}{\rho + k}$$

$$l_{11} = \frac{w_2}{\rho}\left[\left(1-\varepsilon\right)a - bw_2 + \left(p + \eta w_2\right)\beta^2 - d\left(\alpha + d\right)w_1 - \frac{\mu d w_1 \lambda}{\rho + k}\right] + \frac{1}{2\rho}\left[w_2\left(\alpha + d\right) + \mu\frac{w_2 \lambda}{\rho + k}\right]^2$$

$$x_4 = \frac{\lambda p}{\rho + k}$$

$$y_4 = \frac{\lambda\left(p + \eta w_2\right)}{\rho + k}$$

$$l_{12} = \frac{1}{\rho}\left\{\begin{array}{l} p\left[a + \left(\alpha^2 + \alpha d - b + \dfrac{\lambda\alpha\mu}{\rho + k}\right)\left(w_1 + w_2\right)\right] + \beta^2\left(p^2 + p\eta w_2 + \dfrac{1}{2}\eta^2 {w_2}^2\right) \\ +\eta w_2\left[\left(1-\varepsilon\right)a - bw_2 + w_2\left(\alpha + d\right)^2 + \mu\dfrac{w_2\lambda}{\rho + k}d\left(\alpha + d\right)w_1 + \dfrac{\lambda\mu d}{\rho + k}\left(w_2 - w_1\right)\right] \\ \dfrac{\lambda\mu}{\rho + k}\left[pw_1 + \left(p + \eta w_2\right)w_2\right]\left(\alpha + d + \mu\dfrac{\lambda}{\rho + k}\right)^2 \end{array}\right\}$$

End of proof.
